# Novel genes associated with lymph node metastasis in triple negative breast cancer

**DOI:** 10.1038/srep15832

**Published:** 2015-11-05

**Authors:** Andrea Mathe, Michelle Wong-Brown, Brianna Morten, John F. Forbes, Stephen G. Braye, Kelly A. Avery-Kiejda, Rodney J. Scott

**Affiliations:** 1 Centre for Information Based Medicine, Hunter Medical Research Institute, NSW, 2305, Australia; 2 Priority Research Centre for Cancer, School of Biomedical Sciences and Pharmacy, Faculty of Health, University of Newcastle, NSW, 2308, Australia; 3 Dept. of Surgical Oncology, Calvary Mater Newcastle Hospital; Australian New Zealand Breast Cancer Trials Group; and School of Medicine and Public Health, University of Newcastle, NSW, 2308, Australia; 4 Hunter Area Pathology Service, John Hunter Hospital, Newcastle, NSW, 2305, Australia

## Abstract

Triple negative breast cancer (TNBC) is the most aggressive breast cancer subtype with the worst prognosis and no targeted treatments. TNBC patients are more likely to develop metastases and relapse than patients with other breast cancer subtypes. We aimed to identify TNBC-specific genes and genes associated with lymph node metastasis, one of the first signs of metastatic spread. A total of 33 TNBCs were used; 17 of which had matched normal adjacent tissues available, and 15 with matched lymph node metastases. Gene expression microarray analysis was used to reveal genes that were differentially expressed between these groups. We identified and validated 66 genes that are significantly altered when comparing tumours to normal adjacent samples. Further, we identified 83 genes that are associated with lymph node metastasis and correlated these with miRNA-expression. Pathway analysis revealed their involvement in DNA repair, recombination and cell death, chromosomal instability and other known cancer-related pathways. Finally, four genes were identified that were specific for TNBC, of which one was associated with overall survival. This study has identified novel genes involved in LN metastases in TNBC and genes that are TNBC specific that may be used as treatment targets or prognostic indicators in the future.

Triple negative breast cancer (TNBC) is the most aggressive breast cancer subtype. This subtype is characterised by the absence of the estrogen (ER) and progesterone (PR) receptors, and the human epidermal growth factor receptor 2 (HER2/HER neu). Further characteristics of the TNBC subtype are: a high proportion of germline *BRCA1* mutation carriers present with TNBC, high mitotic counts, and a high proportion of somatic p53 mutations^1^. TNBC accounts for somewhere between 10% and 17% of all breast cancers and is more frequent in women diagnosed at younger ages (under 40 years of age/pre-menopausal^2^), and those of African-American descent^3^. Women with TNBC have a poorer prognosis with an increased number and earlier appearance of metastatic disease (on average within the first 2.6 years after initial diagnosis^4^) compared to other breast cancer subtypes^1^. Metastatic disease is the most common cause for death from this cancer^5^,^6^,^7^.

The absence of the three receptors significantly reduces targeted treatment options for patients with TNBC and the only treatment remains radiotherapy and chemotherapy^3^. There are several trials with newer disease specific agents that include, poly (ADP-ribose) polymerase (PARP) inhibitors, angiogenesis inhibitors, EGFR-targeted agents, src kinase inhibitors, an androgen receptor inhibitor (bicalutamide), epigenetic targeting, and PI3K-pathway inhibition^1^,^8^. None of these trials have thus far been associated with any significant improvement in TNBC patients. Thus, new treatment targets are urgently needed for women diagnosed with this breast cancer subtype. Taken together the evidence indicates that TNBC is a heterogeneous disease where for example PARP-inhibitors demonstrate good effects in *BRCA1* mutation-positive patients diagnosed with TNBC, but not in other TNBC patients^9^. This effect can be explained by the importance of both genes (*BRCA1* and PARP) in DNA repair, such that if both are functionally inactive DNA damage will not be repaired and the affected cell dies^9^. There is a paucity of studies that have focused on identifying molecular biomarkers that are predictive of metastatic disease in TNBC patients.

microRNAs (miRNAs) are small (18–21 nucleotides) non-coding RNAs, which are able to alter gene expression post-transcriptionally. microRNAs have been found in multiple species, where they are highly conserved, suggesting they play a universal role in the regulation of gene expression. miRNAs regulate multiple biological processes including proliferation, cell death, development, genomic stability, and EMT^10^,^11^—key processes in tumour development identified by Weinberg and Hanahan. They not only regulate normal physiological change but are also involved in pathological conditions, such as cancer^12^.

There are multiple studies that have shown altered miRNA levels in TNBC patients compared to healthy controls and compared to other breast cancer subtypes^13^,^14^,^15^,^16^. At present there is little known about the influence of miRNAs and metastasis in TNBC, or what genes they control to regulate this process. In this study we have correlated microRNA expression analysis^16^ with gene expression in a series of TNBC patients where we were able to examine tumour tissue, normal adjacent tissue and lymph node metastases from the same patient and compared that to tumours derived from patients who did not have metastases.

A recent study by Cascione *et al.* (2013) identified a number of genes as biomarkers that are associated with survival in TNBC using a series of breast tumours and matched lymph node metastases^17^, however they only examined 230 cancer-related mRNAs, whereas in the current study a total of 11,000 lncRNAs, 24,000 genes and 30,000 coding transcripts were examined. Using this approach we were able to better define gene differences between primary tumours without disease spread and tumours that had lymph node involvement. Especially important are the findings associated with non-coding transcripts (like microRNAs and long non-coding RNAs) that are predicted to play an important role in cancer progression^18^,^19^. In this study we have identified a gene expression profile in a series of TNBC patients where we were able to examine tumour tissue, normal adjacent tissue and lymph node metastases from the same patient and compared that to tumours derived from patients who did not have any metastatic disease. We have identified TNBC-specific transcripts for tumour versus normal tissue that has been validated in an independent TNBC cohort as well as the *The Cancer Genome Atlas* (TCGA) TNBC breast cancer cohort^20^. Further, we analysed the relationship of these genes with overall survival. Finally, we were able to correlate the gene expression changes from this study with a previous microRNA study using the same breast cancer samples to confirm the relationship between microRNAs and lymph node metastases^16^.

## Results

### Differentially expressed genes in all IDC compared to NAT samples

We compared the gene expression of 33 grade 3 primary invasive ductal carcinomas (IDCs) to 17 normal adjacent tissue (NAT) samples (Supplementary Table 1) to reveal gene and lncRNA transcripts significantly associated with TNBC. A total of 185 genes were revealed to have significantly altered expression in tumour samples compared to NAT samples. Unsupervised hierarchical clustering was performed, which showed clear clusters between these two groups (IDC versus NAT). Within the 185 differentially expressed genes 90 were up-regulated in all tumour samples and 95 down-regulated (Supplementary Figure 1A and Supplementary Table 2).

We used the Ingenuity Pathway analysis tool (IPA) to study gene networks and pathways that involve the genes of interest. All 185 significant genes, with their expression values from the tumour versus normal comparison were used to perform a Core Analysis. This analysis was used to interpret the dataset in the context of biological processes that included pathways, upstream regulators and molecular networks. The analysis revealed that the disorder with the highest number of affected genes was Cancer with 119 out of 185 genes. Multiple genes were involved in the process of Cellular Development (60 genes) and Tissue Development (56 genes). The top upstream regulators of our genes of interest are *PTGER2, ERBB2, E2F4*, and *TGFB1* (Supplementary Table 3).

To validate the genes from the tumour versus normal comparison we used an independent cohort containing 16 TNBC samples, 4 NAT and 48 samples of other breast cancer subtypes (Supplementary Table 4). We compared the expression of the 185 differentially expressed genes from our discovery cohort in all TNBC cases (n = 16) to the 4 NAT samples in our second cohort. This validation substantiated the role of 99 of the 185 genes (only 152 of them were on the array that was used for the validation cohort; 99 = 65.13%) that were differentially expressed in the two TNBC cohorts. Additionally, we performed a further validation using the TCGA breast cancer cohort. We compared tumour vs normal samples from 55 TNBC cases and 5 matched normal cases, which validated 95 of the initial 185 significant genes. By comparing all data sets, we identified 66 genes that were differentially expressed in TNBC in each of the cohorts (Table 1). Unsupervised hierarchical clustering of these results is shown in Supplementary Figure 1 A (185 genes in study cohort) B (66 genes in study cohort), C (66 genes in second cohort), D (66 genes in TCGA cohort).

### Identification of TNBC specific genes

We used our second cohort and the TCGA breast cancer cohort to identify which of the validated genes in each cohort are specific to TNBC. Therefore we performed tumour vs normal comparisons in all non-TNBC cases in both cohorts and compared this to differentially expressed genes in the TNBC cohorts. The results from this analysis are summarised in Table 2.

**Figure 1:**
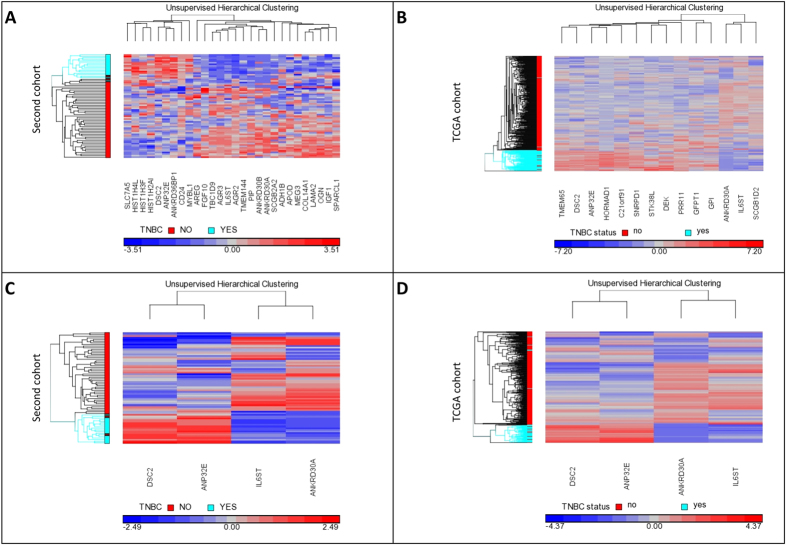
Unsupervised hierarchical clustering of TNBC specific genes in the second cohort and the TCGA cohort. Through analysis of our second cohort (48 non-TNBC, 16 TNBC samples) we identified 28 TNBC specific genes, which were used for unsupervised hierarchical clustering of TNBC and non-TNBC samples (**A**). The TCGA breast cancer cohort (313 non-TNBC, 55 TNBC samples) identified 14 TNBC specific genes. The unsupervised hierarchical clustering of the 14 TNBC specific genes successfully clustered the TCGA breast cancer cohort in TNBC patients and non-TNBC patients (**B**). There are four common TNBC specific genes in both cohorts (our second cohort and the TCGA breast cancer cohort) – these four genes are *ANKRD30A, ANP32E, DSC2, IL6ST*. (**C**) shows the unsupervised hierarchical clustering of these four genes in the second cohort and (**D**) shows the clustering of these genes in the TCGA breast cancer cohort. TNBC samples are shown in light blue and non-TNBC samples are shown in red in the sample tree on the left (y-axis). Genes are clustered along the x-axis. Low gene expression is shown in blue, high expression is shown in red and equivocal expression is shown in grey.

By performing an IDC versus NAT comparison in our second cohort for all non-TNBC (n = 48) samples and comparing that to the list of genes in the TNBC set (n = 16) we found that, from the 99 validated genes, 28 were found to be differentially expressed in TNBC cases compared to non-TNBC cases, we will refer to these as TNBC specific genes (Fig. 1A; and Supplementary Table 2). Figure 1A shows distinct clustering of the TNBC samples compared to the non-TNBC samples in our second cohort, based on the expression of the 28 TNBC specific genes.

Further to this, the same analysis was done in the TCGA breast cancer cohort (excluding TNBC samples). We compared the gene expression of 313 non-TNBC cases and 43 matched normal samples from the TCGA breast cancer cohort, this was then compared to the 95 validated genes from the TNBC TCGA cohort. In this analysis, we identified that 14 of the 95 validated genes were differentially expressed solely in the TNBC cohort and not in non-TNBC cases and were hence TNBC specific. Figure 1B shows unsupervised hierarchical clustering of the 14 TNBC specific genes in the TCGA cohort.

Finally, by comparing the genes that were found to be TNBC specific genes from our second cohort and the TCGA cohort, we found that four of them (*ANKRD30A, ANP32E, DSC2, IL6ST*) were common to both TNBC cohorts (Fig. 1). Figure 1C,D show the unsupervised hierarchical clustering of the four validated TNBC specific genes in our study cohort, our second cohort and the TCGA cohort. As can be seen in this analysis, *IL6ST* and *ANKRD30A* were expressed at lower levels in TNBC when compared to non-TNBC, while *ANPE32E* and *DSC2* were expressed at higher levels in TNBC when compared to non-TNBC.

### TNBC-specific genes associated with overall survival

**Figure 2:**
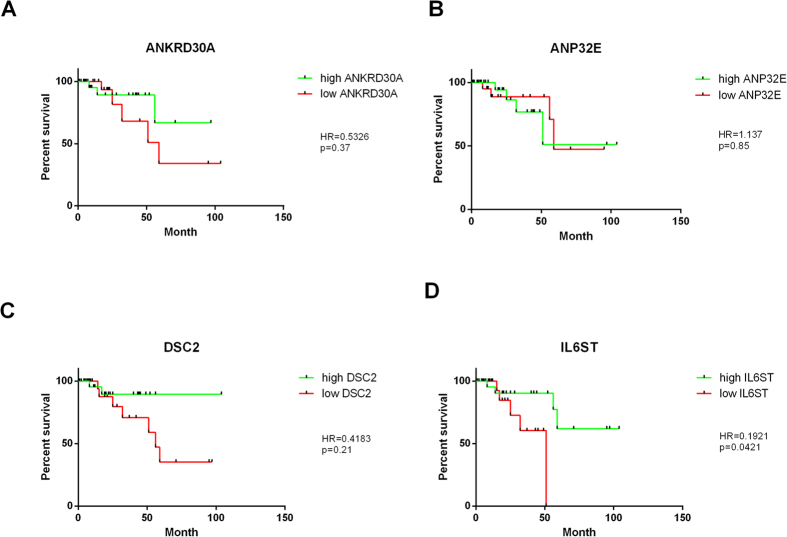
Kaplan-Meier analysis of four validated TNBC specific genes. Kaplan-Meier analysis was performed on the four validated TNBC specific genes (*ANKRD30A, ANP32E, DSC2, IL6ST*). The analysis of (**A**) *ANKRD30A* in TCGA TNBC cohort, Hazard ratio (HR) = 0.5326; p = 0.37. (**B**) *ANP32E* in TCGA TNBC cohort; HR = 1.137; p = 0.85. (**C**) *DSC2* in TCGA TNBC cohort; HR = 0.4183; p = 0.21. (**D**) *IL6ST* in TCGA TNBC cohort; HR = 0.1921; p = 0.0421*. The Hazard ratio (HR) is smaller than 1 for three genes, which means down-regulated genes decrease overall survival in TNBC, whereas up-regulated genes would increase overall survival. A significant correlation to overall survival can be seen for *IL6ST* (p < 0.05). The p-value shows the significance of the difference between the survival curves.

We used the breast cancer cohort from *the Cancer Genome Atlas* (TCGA) which contains 55 TNBC samples, of which nine patients died and 46 are still alive. With this information we were able to determine if our 4 TNBC specific genes (*ANKRD30A, ANP32E, DSC2, IL6ST*) were associated with survival. We performed Kaplan-Meier-Analysis on the four genes that are TNBC specific in our second cohort as well as in the TCGA cohort (*ANKRD30A, ANP32E, DSC2, IL6ST*). The results from this analysis can be seen in Fig. 2. The survival curves from Fig. 2 show that two of the genes (*ANKRD30A, DSC2*) show a non-significant trend that low gene expression is associated with shorter survival, *ANP32E* expression shows no association to survival. High expression of *IL6ST* shows significant (p = 0.0421) association with better survival outcomes.

### Genes associated with lymph node metastasis

Gene expression analysis of the 33 primary TNBC tumours, 15 matched lymph node metastases, and 17 matched normal breast tissues was undertaken to identify which transcripts were associated with various aspects of TNBC. The arrays were analysed using Genomic Suite 6.6 (Partek).

**Figure 3:**
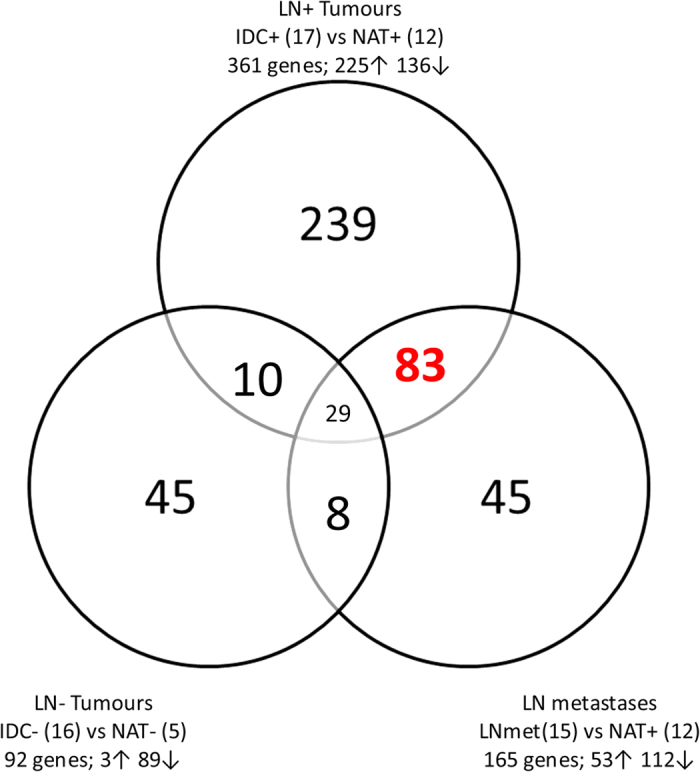
Venn Diagram for identification of genes associated with lymph node metastasis. All samples are categorised in lymph node positive primary tumours (IDC+), lymph node negative primary tumours (IDC−), lymph node metastases (LNmet) and matched normal adjacent tissue for lymph node positive cases (NAT+) and lymph node negative cases (NAT−). Gene expression was analysed in all three tumour categories compared to their matched NAT samples. Highlighted in red are the 83 genes that are differentially expressed in IDC+ and LNmet samples but not in IDC− samples.

To identify genes responsible for metastasis we performed a categorical comparison. We compared gene expression in three different categories. Category **1**: lymph node positive primary tumours (IDC+) versus matched normal adjacent tissue **(IDC+ vs NAT+)**, category **2**: lymph node negative primary tumours (IDC-) versus matched normal adjacent tissue **(IDC− vs NAT−)**, and category **3**: lymph node metastases versus matched normal adjacent tissue **(LNmet vs NAT+)** (Fig. 3). The focus of this study is common genes of category 1 and 3, as these genes may be biomarkers for metastatic spread.

We identified 361 genes that were differentially expressed in IDC+ tissue compared to matched NAT (category 1), 92 were differentially expressed in IDC- samples compared to their matched NAT (category 2), and 165 had significantly altered expression in LNmet samples compared to matched NAT samples (category 3). There were 83 genes that were specifically associated with LN metastasis, since they were differentially expressed in category 2 and 3. This included 72 protein coding genes and 11 non-coding RNAs (such as MEG3); out of these 36 had increased expression in IDC+ and LNmet samples compared to NAT+ samples, and 47 showed decreased expression in IDC+ and LNmet samples compared to NAT+ samples (Supplementary Table 5).

A second Core analysis was performed in IPA with the 83 significant genes that were potential biomarkers for metastasis. Similarly the top candidate for *diseases and disorders* was *Cancer*, with 62 genes involved. The majority of these are involved in cellular processes like cell cycle, DNA replication, recombination, repair, and cell death/survival (Supplementary Table 6). Looking at the upstream regulators of the genes in IPA, we identified a number of activated genes (*CSF2, PTGER2, FOXO1* - mostly involved in proliferation, differentiation and invasion) and inhibited genes (*TP53, TCF3* - tumour-suppressor, apoptosis regulator). None of these genes were present in the validated TNBC specific gene list but 63% (52 genes) were differentially expressed in tumour versus normal comparisons when all cases were compared in cohort 2 and the TCGA cohort indicating that they may be important biomarkers of metastatic progression in all breast cancers. We also performed survival analysis (using the complete TCGA breast cancer cohort) of the five most up- and five most down-regulated genes from this signature, but were unable to show a significant association to overall breast cancer survival (data not shown).

Our previous study identified miRNAs associated with LN metastasis^16^. We performed a correlation study (see Material and Methods) to identify microRNAs that could potentially regulate the LN metastasis-associated genes identified in this study. Twenty two microRNAs were found to be significantly altered in lymph node positive primary tumours (IDC+) and lymph node metastases (LNmet) but not in lymph node negative primary tumour (IDC−) samples (Supplementary Table 7). Their expression was correlated with the 83 gene biomarkers using Genomic Suite 6.6 (Partek). The correlation analyses revealed that 17 of these microRNAs can potentially target 50 of the 83 genes. Several genes (31) can be targeted by multiple microRNAs (Supplementary Table 8).

## Discussion

The outcome for triple negative breast cancer (TNBC) patients is generally considered to be poor. Nevertheless, TNBC is a heterogeneous disease and the probability of survival and/or relapse is different from patient to patient. Specific gene expression patterns can be used to identify differences in patients with phenotypically similar disease but with very different disease outcomes^21^,^22^. The current study aimed to identify expression differences associated with metastatic disease in TNBC. Other reports have provided gene expression profiles for TNBC, but most did not include any comparison to normal adjacent tissue, nor did they separate primary IDCs from lymph node metastases^23^,^24^. Other reports identified prognostic markers in TNBC by comparing gene expression profiles of TNBC patients to all other non-TNBC samples^25^,^26^, which can be used to assess the general risk of relapse, but not sufficiently specific to identify biomarkers associated uniquely metastatic spread in TNBC. The study herein confirms the more aggressive nature of TNBC and provides an insight into which pathways are most important in differentiating women at risk of relapse. Furthermore, this study used NAT, IDC and LNmet samples that were biopsied at the same time, this allowed the identification of differences in these three tissue types without the influence of further disease progression. Due to the structure of this analysis the criteria meant only a small sample cohort of patients could be identified, but allowed us to reveal significant biological differences between the three different tissue types. This allowed us to examine actual differences between TNBCs that had begun to move away from their primary location compared to those that had not. This is in contrast to studies aimed at identifying differences between distant metastatic lesions that may have been influenced by other tissue specific factors and an increased mutation burden.

In this study we identified and validated TNBC-specific genes, which we were able to associate with overall survival. Further to this, we have revealed genes associated with lymph node metastasis in TNBC and showed that these are potentially targetable by microRNAs with altered expression. This included the lncRNA gene *MEG3,* which has not previously been reported to be associated with metastatic breast cancer.

We compared IDC to NAT samples to identify tumour-associated genes in TNBC. Although, it has been shown that normal tissue adjacent to TNBC tissue has altered gene expression compared to healthy controls, and that NAT samples already show an expression profile indicating DNA repair deficiency^27^,^28^, we reasoned that by comparing NAT to IDC samples we would identify further changes in tumour progression.

The 185 genes that we identified showed clear hierarchical clustering of NAT samples and IDC samples. The pattern included 90 genes that were significantly up-regulated in all tumour samples and 95 that are significantly down-regulated.

We were able to confirm the expression pattern of 66 of the 185 genes in our two validation cohorts (cohort 2 and TCGA), which also allowed us to reveal four genes that are specific for TNBC, by comparing the expression of the validated genes to significantly altered genes in a tumour versus normal comparison in other breast cancer subtypes (Fig. 1 and Supplementary Figure 1).

These four TNBC specific genes are *ANKRD30A, ANP32E, DSC2, IL6ST* (Fig. 1, Table 1). The expression of these four genes alone clearly clustered TNBC from non-TNBC tumours. Together with the survival data from the TCGA cohort we were able to perform Kaplan-Meier analysis (Fig. 2). Two of the four genes (*ANKRD30A, DSC2*) show a non-significant trend that higher gene expression is associated with longer survival time. *ANP32E* does not show any association with overall survival in TNBC patients. Finally, higher expression of *IL6ST* shows significant association with longer overall survival in TNBC patients. To the best of our knowledge *IL6ST* has not been associated with overall survival in TNBC before. Nevertheless, it has been shown to be associated to ER expression and with TNBC development/progression^29^,^30^,^31^.

*ANKRD30A* is well known in breast cancer as NY-BR-1 (Breast Cancer Antigen NY-BR-1)^32^, nevertheless it has not yet been associated as a TNBC specific marker. Jaeger *et al.* identified the gene in 2001 as a tissue-specific putative transcription factor in breast tissue by serological screening of a breast cancer library^33^. It has been found to be highly expressed in the majority of breast cancer cases, but our studies have shown that it has significantly lower expression in TNBC than in the other breast cancer subtypes. Even though our survival data for this gene did not show a significant effect, there is a non-significant trend that lower gene expression appears to be associated with worse overall survival. *ANKRD30A* has been suggested as a potential target for immunotherapy due to its highly restricted expression pattern in breast cancer it may function as a breast differentiation antigen^34^. It has also been shown that high *ANKRD30A* expression is associated with lymph node negativity, as well as with the expression of HER2 and ER^35^, which supports our findings of lower expression in TNBC and supports the non-significant trend we identified that those with the lowest expression had worst survival.

*ANP32E* was more highly expressed in TNBC than non-TNBC in our study and has recently been identified as a histone chaperone that removes H2A.Z from chromatin^36^. H2A.Z is a histone variant which plays a crucial role in various key processes like DNA repair^37^ and cancer initiation and progression^38^,^39^. Together with *DSC2,* it is part of a six-gene signature predicting breast cancer lung metastasis^40^. Later it has been shown that these two genes are associated with breast cancer subtype (with ER-negative breast cancers, basal-like breast cancers)^41^, but neither of them have been stated as TNBC specific markers. *DSC2* is a Ca^2+^-dependent transmembrane cell adhesion protein of desmosomes^42^. Except for the mentioning in the six-gene signature by Landemaine *et al.*, the function of *DSC2* has not been studied in breast cancer.

*IL6ST* is the signal transducer for interleukin 6 (IL6), ciliary neurotrophic factor (CNTF), leukemia inhibitory factor (LIF), and oncostatin M (OSM). We showed that *IL6ST* was lower in TNBC when compared to non-TNBCs. High expression of *IL6ST* has been shown to be a good prognostic factor in breast cancer as it increases patients overall survival^43^, which supports our finding in TNBC where higher expression of IL6ST was shown to be associated with significantly increased survival. Multiple studies identified *IL6ST* as being positively associated with ER-α expression in breast cancer^30^,^31^, which again confirms our findings of decreased levels of this gene in TNBC patients.

The second aim of this study was to identify biomarkers associated with metastasis in TNBC. Lymph node metastases are the first sign of metastatic potential. Even though they are not necessarily associated with prognosis, they provide an ideal tissue to gain knowledge about the mechanisms of tumour progression. In this study, we identified genes that are only differentially expressed in the lymph node positive primary tumours and lymph node metastases (NAT+ v IDC+ and NAT+ v LNmet). By doing so, we identified a unique gene pattern of 83 genes that may be potential biomarkers for metastasis in TNBC. The most significantly down-regulated gene in this comparison was *APOD*, which is a lipocalin. *APOD* is used as a biomarker indicating good prognosis in colorectal cancer, if its expression level is increased since *APOD* induces growth arrest and reduces cell proliferation when its levels are elevated^44^,^45^,^46^. It is known to interact with ERα and can function as a Tamoxifen transporter, which suggests co-expression of ERα and APOD would predict a better outcome^47^. The fact that its expression is significantly decreased in our cohort (LNmet vs NAT+ fold change = −9.46; IDC+ vs NAT+ fold change = −6.62) confirms the poor prognosis for TNBC patients, especially patients with metastases.

We identified 72 protein coding genes and 11 non-coding RNA (ncRNA) to be associated with lymph node metastasis. The discovery that not just changes in protein coding genes but also in non-coding genes are responsible for cancer development and cancer progression has transformed cancer research in recent years^48^. The majority of ncRNA species remains to be discovered and many of known ncRNAs still have not been assigned a function. One of the first lncRNAs to be associated with breast cancer survival was *HOTAIR*^49^ and as such we have shown that a second lncRNA (MEG3) is also associated with LN metastases further supporting the important role of lncRNAs in disease. *MEG3* is known to be down-regulated in multiple cancers and tumour cell proliferation is inhibited by *MEG3* expression. It is able to alter the *p53* pathway and acts as a tumour suppressor^50^,^51^. Recently it has been discovered that *MEG3* overexpression leads to G2/M cell cycle arrest and apoptosis, which would explain increased cell growth when *MEG3* is down-regulated^52^. In bladder cancer *MEG3* activates autophagy, which leads to increased cell proliferation^53^. So far the function of *MEG3* in triple negative breast cancer is unknown and needs further investigation.

In our previous study we used this TNBC cohort to identify microRNAs (miRNAs) that were associated with lymph node metastasis^16^. To the best of our knowledge, there has not been a study to correlate altered microRNA expression to gene expression changes in the same cohort, including the comparison of lymph node metastases to primary tumours in TNBC. The inclusion of expression values into the target prediction algorithms within 2 software suites (TargetScan 6.2, microcosm) increases the biological relevance of the prediction. In our cohort, 17 significantly altered microRNAs are likely to contribute to the identified gene expression profile of 83 gene biomarkers for metastasis (Supplementary Table 8). The only other integrated microRNA and mRNA study in a pure TNBC cohort compared expression changes from lymph node metastases to normal adjacent tissue, and primary tumours to normal adjacent tissue, without separation of lymph node positive and lymph node negative tumours and only looking at a set of 230 genes^17^. Nevertheless we can confirm 6 of their identified genes (*SPP1, TOP2A, AREG, EGR1, CD34*, and *IGF1*) as well as one of the identified miRNAs (hsa-miR-101), that they found to be differentially expressed in lymph node metastases compared to primary tumours and to normal tissue. Another miRNA-mRNA integration study in breast cancer by Buffa *et al.* found three miRNAs (hsa-miR-324, hsa-miR-27b, and hsa-miR-150) to be associated with progression pathways in TNBC^54^, however this study did not integrate these miRNAs with genes in TNBC alone and did not perform a comparison to normal tissue nor to lymph node metastases. These miRNA were not found to be differentially expressed between tumour versus normal tissue in our analysis and were therefore excluded from further analysis. A number of our miRNAs of interest have been associated with TNBC or tumour progression previously (summarised in Supplementary Table 9). As an example miR-205 is known to be down-regulated in TNBC^55^, it is a known tumour-suppressor-miR that targets E2F1, LAMC1, suppresses cell proliferation, cell cycle and tumour growth^56^. Most interesting for this study miR-205 has also recently been associated to lymph node metastasis in a study by Berber *et al.*^57^.

We were unable to associate the five most up- and five most down-regulated genes to overall survival in the TCGA cohort. Nevertheless, there are a number of genes within our list of 83 genes, associated to lymph node metastasis, that have been associated with prognosis in other studies. *TOP2A* is the gene with the highest fold change in our cohort (LNmet vs NAT+ fold change = 4.628; IDC+ vs NAT+ fold change = 5.08). This gene encodes an enzyme that is important for chromosome condensation, chromatin separation during DNA replication. Over-expression of this gene is a known prognostic marker for poor outcome in several malignancies, including TNBC^58^,^59^,^60^. Even though it has been shown that high expression of *TOP2A* is associated with worse prognosis, it also provides knowledge about the efficacy of treatment since TNBC patients with high *TOP2A* expression respond better to anthracycline therapy^58^.

A recent study performed a meta-analysis of global gene expression profiles of TNBC to identify genes of prognostic value^61^. This study was only looking at over-expressed genes, to identify potential treatment targets. They identified the majority of altered genes in TNBC are involved in chromosomal instability (CIN) and ER signalling. Especially genes causing CIN often lead to aneuploidy in several cancer types^62^. In general overexpression of these genes and aneuploidy are associated with poor outcome. These studies provided lists of genes correlated with aneuploidy, in which 16 of the genes we identified also appear^62^,^63^. These genes are: *TPX2, TOP2A, UBE2C, MELK, RAD51AP1, MAD2L1, ATAD2, CKS2, ECT2, GPI, STAT1, CXCL9, CXCL10, CXCL11, SMS*, and *TNFRSF13B*. All of the genes are up-regulated in our cohort, suggesting a poor outcome for the patients and possible aneuploidy. The study from Carter *et al.* (2006) shows that the gene with the highest level of consistent correlation with total functional aneuploidy is *TPX2* which has been supported by Szasz *et al.* (2013) who used a gene set of 4 CIN genes to measure tumour aneuploidy, two of them are *TOP2A* and *TPX2*^64^. Both genes are significantly up-regulated in our cohort, confirming the relationship between aneuploidy and poor outcome for patients with TNBC.

In conclusion, we identified TNBC-specific gene expression profiles and showed novel associations for TNBC-specific genes to prognosis. Additionally, we identified a gene expression profile for genes associated with lymph node metastasis, which we were able to correlate our previous study results of microRNA expression profiles with the profiles of this study. In this way we identified novel microRNA and mRNA interactions, supporting important pathways in TNBC development.

## Material and Methods

### Study design

A total of 33 grade 3, triple negative, invasive ductal carcinomas (IDCs), 17 matched normal adjacent tissues (NAT), and 15 lymph node metastases (LNmet) were used for gene expression microarray analysis. All samples were formalin-fixed, paraffin-embedded (FFPE) and obtained from the archives of the Hunter Area Pathology Service, John Hunter Hospital, Newcastle, Australia. This cohort has been described previously^16^. The triple negative phenotype, areas of NAT, invasive cancer and LNmets were confirmed by a pathologist. Further we show significantly lower ER, PR and HER2 expression in the TNBC samples compared to receptor-positive patients in supplementary Figure 2. A 1.5 mm punch biopsy was used to isolate these sections, performed as described previously^16^. Areas of NAT, IDC and LNmet tissue were biopsied at the same time to examine genetic differences in these three tissue types without the influence of time/further disease progression. The pathologist confirmed that the tumour volume in the core biopsy was >70% of the total core. This study complies with the Helsinki Declaration with ethical approval from the Hunter New England Human Research Ethics Committee (Approval number: 09/05/20/5.02). A waiver of consent was granted for this study in accordance with the *National Statement on Ethical Conduct in Research Involving Humans*.

### RNA extraction

The RNA extraction of all samples was performed as previously described^16^.

### Gene expression microarrays and analysis

The array results have been deposited in Gene Expression Omnibus (GEO) with Accession No. GSE61725.

100 ng total RNA of all FFPE samples was amplified (Ovation FFPE WTA kit) and biotinylated (Encore Biotin module) according to the manufacturers’ instructions (Nugen, San Carlos, California, United States). The samples were hybridised to HuGene 2.0 arrays (Affymetrix, Santa Clara, California, United States) and 17 hours later washed and stained. The Arrays were scanned on a GeneChip Scanner 3000 7G (Affymetrix).

The HumanGene 2.0 arrays (Affymetrix) contain probe features representing 11,000 lncRNAs, 24,000 genes, and 30,000 coding transcripts. The data was imported to Genomic Suite 6.6 (Partek) and a robust multi-array analysis (RMA) was performed, which included log2 transformation, background correction, quantile normalisation and summarisation of the probe features resulting in a set of expression signal intensities.

Unsupervised hierarchical clustering was performed on genes that were found to be significantly different in the comparison of all IDC vs all NAT samples (p-value < 0.5; fold change>1.5 or < − 1.5). Correction for multiple testing was performed using Benjamini–Hochberg procedure.

We compared gene expression in three different categories. Category 1: invasive ductal carcinoma with lymph node metastases versus normal adjacent tissue from patients with lymph node metastases (IDC+ vs NAT+), category 2: IDC- vs NAT-, and category 3: lymph node metastases (LNmet) vs NAT+ (Fig. 3) (“+” = lymph node involvement; “−” = only a primary tumour). The focus of this study was on common genes of category 1 and 3, proposing to be biomarkers for metastasis.

### Validation of differentially expressed genes

To validate results of the gene expression arrays, we used an independent cohort with 16 TNBC primary tumours and 4 normal adjacent tissues, and 48 IDCs from patients with breast cancer other than TNBC, all samples were fresh frozen tissue cores. Lymph node metastases were not available from these samples, nevertheless 5 of the TNBC cases had known lymph node involvement. These tumour samples were provided by the Australian Breast Cancer Tissue Bank (Darcy Rd, Westmead, NSW, Australia). This study complies with the Helsinki Declaration with ethical approval from the Hunter New England Human Research Ethics Committee (Approval number: 09/05/20/5.02). A waiver of consent was granted for this study in accordance with the *National Statement on Ethical Conduct in Research Involving Humans*.

The samples and the method of RNA extraction have been described previously^65^. The RNA was hybridised to HumanGene 1.0 arrays (Affymetrix) and scanned on a GeneChip Scanner 3000 7G (Affymetrix). The data was imported to Genomic Suite 6.6 (Partek) and analysed as above. Due to the lack of samples from lymph node metastases, only the comparison from tumour versus normal samples could be performed and compared to the previous analyses.

For further validation of the tumour vs normal comparison as well as the identification of TNBC specific genes we used the The Cancer Genome Atlas (TCGA) breast cancer cohort gene expression data^20^. This data contains raw expression values for 55 TNBC samples with 5 matched NAT samples, and 313 non-TNBC samples with 43 matched NAT samples. The data was imported to Genomic Suite 6.6 (Partek) and analysed as above.

### Correlation of genes to overall survival

We performed Kaplan-Meier analysis on the validated TNBC specific genes that we identified. Therefore we used the median gene expression to divide the patients into high and low expression and together with the TCGA survival data we were able to perform the analysis using GraphPad Prism 6 survival analysis tool. P-values were calculated using the log-rank (Mantel-Cox) method and hazard ratios were calculated by the Mantel-Haenszel method.

### Pathway analysis

Differentially expressed transcripts were imported with their expression values into Ingenuity Pathway (IPA), where a Core Analysis was performed to identify their involvement in biological processes, pathways and molecular networks.

### Correlation to microRNAs

To determine if altered microRNA expression contributed to the identified gene expression profiles, we correlated differentially expressed genes to microRNA microarray expression data from the same sample cohort, a previously published study^16^.

Partek Genomics Suite 6.6 was used to identify microRNAs that were differentially expressed in lymph node metastases and lymph node positive primary tumours but not in lymph node negative tumours, compared to matched NAT samples. Significance was reached with p-values <0.05 and two-sided fold change >1.5, these parameters identified 22 microRNAs (see Supplementary Table 7). Correction for multiple testing was performed using Benjamini–Hochberg procedure.

These microRNAs were correlated to significantly altered genes identified in this study. The correlation was performed in Partek Genomic Suite 6.6. Pearson Correlation and Spearman’s rank correlation were used to correlate microRNAs with potential target genes. These correlation analyses include the microRNA and gene expression values as well as two target prediction software suites (TargetScan 6.2, and Microcosm).

### Statistical analysis

Student’s *t*-test and ANOVA were used to analyse differences between two or more groups. Significance was regarded for p < 0.05 and two-sided fold change >1.5.

## Additional Information

**How to cite this article**: Mathe, A. *et al.* Novel genes associated with lymph node metastasis in triple negative breast cancer. *Sci. Rep.* **5**, 15832; doi: 10.1038/srep15832 (2015).

## Supplementary Material

Supplementary Information

## Figures and Tables

**Table 1:** 66 common significantly altered genes comparing tumour vs normal samples in the study cohort, our second cohort and the TCGA cohort.

Transcript ID	Study cohort		Second cohort		TCGA	
	Fold-Change(yes vs. TNBC normal)	p-value(TNBC status)	Fold-Change(IDC vs. NAT)	p-value(TvN)	Fold-Change(IDC vs. NAT)	p-value(TvN)
ABCA10	−3.07	3.59E–21	−4.69	1.76E–09	−2.71	5.51E–10
ABCA6	−8.96	5.42E–23	−5.01	6.37E–06	−3.83	4.95E–10
ABCA8	−20.18	5.12E–29	−5.41	7.23E–05	−2.58	6.91E–10
ABCA9	−15.16	2.85E–35	−4.55	2.43E–06	−2.54	4.50E–08
AGR2	−13.79	5.62E–49	−20.16	3.97E–05	−2.10	3.56E–06
**ANKRD30A**	−31.96	1.48E–25	−43.09	5.71E–12	−4.91	9.08E–12
ANKRD30B	−11.36	1.11E–17	−13.74	0.000141	−2.24	0.000458
ANLN	8.94	2.52E–56	15.88	2.62E–08	2.56	9.26E–06
**ANP32E**	3.06	5.47E–40	2.58	2.37E–06	2.05	0.000932
APOD	−5.16	1.65E–16	−4.71	0.002127	−7.77	9.23E–09
AREG	−16.35	1.54E–12	−7.97	0.000308	−2.06	2.80E–06
ASPM	19.80	2.17E–71	15.69	5.15E–08	2.97	4.08E–07
ATAD2	3.95	3.05E–32	2.93	0.002838	2.63	2.13E–06
BGN	3.38	1.20E–42	8.53	1.04E–05	2.48	1.21E–05
CALU	3.26	1.72E–27	2.86	3.88E–08	2.19	3.88E–06
CCT3	2.46	2.70E–42	2.39	1.16E–07	2.09	3.04E–06
CENPF	18.38	9.29E–69	14.06	1.36E–08	2.94	6.16E–08
CHML	2.58	1.22E–19	3.72	1.11E–05	2.12	2.98E–05
CKS2	5.13	3.45E–56	4.93	2.83E–05	4.20	6.45E–08
COL14A1	−5.57	4.10E–14	−3.01	0.000568	−3.44	5.00E–09
CXCL10	3.09	8.20E–33	24.83	6.19E–06	4.48	1.19E–05
CXCL11	9.68	9.14E–31	11.91	0.000101	2.03	6.14E–05
CXCL9	3.20	6.32E–17	6.31	0.005079	5.14	1.96E–05
**DSC2**	5.31	1.63E–29	1.89	0.006364	2.38	0.0009
ECT2	4.47	2.82E–34	3.38	0.000117	2.52	4.48E–07
EGR1	−12.87	8.24E–38	−4.73	5.73E–05	−3.08	9.70E–07
FGF10	−10.16	5.14E–16	−3.04	3.45E–05	−2.34	7.78E–11
FN1	4.73	3.88E–48	7.90	2.21E–12	3.94	1.26E–06
FREM1	−13.48	2.05E–36	−2.37	7.86E–06	−2.18	9.51E–09
GPI	1.87	3.91E–09	3.66	0.000226	2.23	9.43E–06
HIST1H2BD	2.60	1.71E–32	2.30	0.004616	2.47	0.00052
HIST1H3F	2.65	1.06E–38	1.61	0.002149	4.64	8.42E–07
IGF1	−5.83	1.42E–18	−3.65	0.000782	−2.96	1.21E–08
**IL6ST**	−3.20	1.92E–23	−3.39	8.60E–08	−2.36	7.53E–06
INHBA	5.23	3.52E–60	7.48	1.49E–06	2.12	3.42E–06
JAM2	−4.20	2.88E–29	−2.47	0.001	−2.08	6.94E–08
KIF11	6.69	6.47E–60	8.69	1.39E–05	2.40	1.92E–06
KPNA2	3.72	6.80E–37	5.28	4.32E–08	2.16	9.43E–05
LAMA2	−4.28	4.96E–14	−2.51	0.001598	−2.02	7.43E–07
LIFR	−7.78	1.84E–43	−5.28	0.000688	−2.67	3.25E–07
MEG3	−3.39	3.52E–68	−1.66	0.003507	−2.53	6.64E–09
MELK	14.34	1.72E–61	14.49	1.27E–07	2.13	2.71E–05
MKI67	14.02	1.35E–51	10.94	5.24E–08	2.18	9.96E–06
MME	−8.64	6.89E–40	−5.17	0.001052	−2.08	6.00E–05
MYBL1	5.34	8.72E–17	4.34	0.005911	2.24	0.000728
NUSAP1	6.35	3.64E–46	8.42	1.55E–06	2.14	7.03E–07
OGN	−58.54	4.14E–32	−5.26	5.37E–05	−4.13	7.52E–11
PDK4	−15.78	4.50E–36	−3.74	0.006427	−2.53	0.0019
PRDX1	1.91	8.91E–14	1.92	0.001102	2.00	2.67E–05
PRR11	2.52	2.88E–17	9.50	1.28E–08	2.30	1.58E–06
RAD51AP1	4.44	1.47E–29	4.30	0.00132	2.20	7.80E–06
RBMS3	−5.25	2.31E–35	−2.82	0.00085	−2.17	3.32E–07
SLC7A5	3.96	2.13E–32	2.25	0.000817	2.09	0.002088
SMC4	3.15	1.08E–37	2.70	7.86E–05	2.01	1.56E–05
SNRPD1	1.91	2.42E–26	2.39	0.000965	2.06	0.000118
SPARCL1	−2.16	2.33E–13	−1.93	0.001856	−2.65	9.09E–06
SPP1	6.68	2.97E–12	11.48	4.91E–06	3.40	2.30E–06
STIL	9.28	1.33E–38	4.23	9.29E–05	2.14	7.21E–06
SULF1	2.95	9.23E–24	5.76	2.21E–07	3.38	7.50E–08
TAT	−3.68	3.73E–15	−60.22	2.49E–09	−2.27	6.83E–06
TBC1D9	−7.35	7.69E–50	−4.12	2.20E–07	−2.25	1.37E–07
TOP2A	8.58	4.63E–44	15.44	4.91E–10	4.13	6.26E–08
TPX2	11.10	2.56E–63	11.83	2.71E–09	3.57	6.46E–08
TSHZ2	−3.71	3.89E–20	−4.92	3.56E–05	−2.28	2.10E–06
UBE2C	19.93	8.26E–57	2.60	0.000417	2.22	1.53E–07
UBE2T	6.22	5.05E–47	8.55	1.24E–07	2.18	2.82E–05

The 4 TNBC specific genes are written in bold.

**Table 2:**
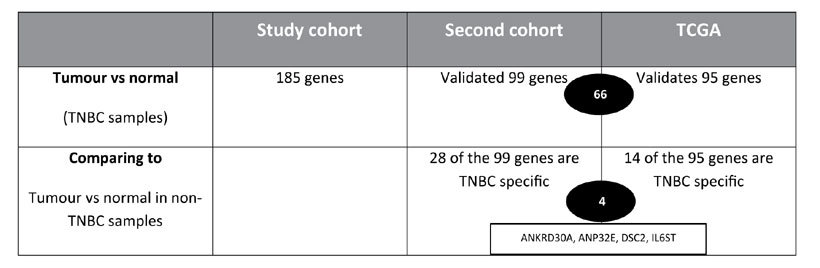
Summary of the gene expression validation in our second cohort and the TCGA TNBC cohort and discovery of TNBC specific genes.

We identified 185 significantly altered genes by comparing all TNBC samples (33) versus all matched normal samples (17) in our study cohort. We then compared all TNBC tumours (16) from our second cohort to all normal samples (4), which validated 99 of the 185 genes. Further, we used the TCGA TNBC cohort to compare 55 TNBC samples to 5 matched normal samples, which validated 95 of our initial 185 genes. By comparing the two sets of validated genes we found 66 common genes. To identify TNBC-specific genes, we compared the 99 validated genes from our second cohort in TNBC samples (16) versus non-TNBC samples (48) and discovered that 28 of these genes are specific to TNBC. In the TCGA cohort we compared the 95 validated genes to the expression of 313 non-TNBC samples, which identified 14 TNBC specific genes. There are 4 TNBC specific genes that are common in both cohorts; these are ANKRD30A, ANP32E, DSC2 and IL6ST.
