# Supplementary material for: Novel genes associated with lymph node metastasis in triple negative breast cancer
Source: Sci Rep. 2015 Nov 5;5:15832. doi: 10.1038/srep15832 (PMC4633580; doi:10.1038/srep15832)
Supplement: Supplementary Information [file srep15832-s1.pdf]

Novel genes associated with lymph node metastasis in triple negative breast cancer.

Journal: Scientific Reports

Andrea Mathe[a,b], Michelle Wong-Brown[a,b], Brianna Morten[a,b], John F. Forbes[b,c], Stephen G. Braye[d], Kelly A. Avery-Kiejda[a,b†] and Rodney J. Scott[a,b,d†*].

1. Centre for Information Based Medicine, Hunter Medical Research Institute, NSW, Australia, 2305
2. Priority Research Centre for Cancer, School of Biomedical Sciences and Pharmacy, Faculty of Health, University of Newcastle, NSW, Australia, 2308.
3. Dept. of Surgical Oncology, Calvary Mater Newcastle Hospital; Australian New Zealand Breast Cancer Trials Group; and School of Medicine and Public Health, University of Newcastle, NSW, Australia, 2308.
4. Hunter Area Pathology Service, John Hunter Hospital, Newcastle, NSW, Australia, 2305.

† These authors contributed equally

* Corresponding author

Supplementary Table 1: Clinical information of samples in the study cohort

patient identifier	Tissue sample	LN status	Age at diagnosis	grade	LN status at 1st surgery
1	IDC	IDC LN-	73	3	0/1
2	IDC	IDC LN-	57	3	No lymph nodes found
3	IDC	IDC LN+	45	3	1/1
	NAT	NAT LN+			
4	IDC	IDC LN+	64	3	1/3
	LN	LN			
5	IDC	IDC LN+	56	3	9/22
	LN	LN			
	NAT	NAT LN+			
6	IDC	IDC LN+	48	3	1/3
	NAT	NAT LN+			
7	IDC	IDC LN+	40	3	1/2
	LN	LN			
	NAT	NAT LN+			
8	IDC	IDC LN+	50	3	1/2
	NAT	NAT LN+			
9	IDC	IDC LN+	47	3	4/13
	LN	LN			
	NAT	NAT LN+			
10	IDC	IDC LN-	48	3	0/4
	NAT	NAT LN-			
11	IDC	IDC LN+	28	3	4/12
	LN	LN			
	NAT	NAT LN+			

12	IDC	IDC LN-	44	3	0/1
	NAT	NAT LN-			
13	IDC	IDC LN-	59	3	0/3
14	IDC	IDC LN+	55	3	23/25
	LN	LN			
15	IDC	IDC LN-	52	3	0/23
16	IDC	IDC LN+	78	3	16/19
	LN	LN			
17	IDC	IDC LN-	52	3	0/2
18	IDC	IDC LN+	47	3	1/14
	LN	LN			
	NAT	NAT LN+			
19	IDC	IDC LN-	50	3	0/19
	NAT	NAT LN-			
20	IDC	IDC LN+	42	3	1/9
	LN	LN			
21	IDC	IDC LN-	84	3	0/4
22	IDC	IDC LN-	45	3	0/4
23	IDC	IDC LN-	61	3	0/12
	NAT	NAT LN-			
24	IDC	IDC LN-	51	3	0/3
25	IDC	IDC LN+	66	3	1/23
	LN	LN			
26	IDC	IDC LN-	36	3	0/1
	NAT	NAT LN-			
27	IDC	IDC LN-	73	3	0/1
28	IDC	IDC LN+	42	3	1/15
	NAT	NAT LN+			
29	IDC	IDC LN-	69	3	0/15
30	IDC	IDC LN+	39	3	1/17
	LN	LN			
	NAT	NAT LN+			
31	LN	LN	78	3	2/18
32	IDC	IDC LN+	65	3	5/26
	LN	LN			
	NAT	NAT LN+			
33	IDC	IDC LN+	55	3	1/17
	LN	LN			
	NAT	NAT LN+			
34	IDC	IDC LN-	80	3	0/8
35	LN	LN	72	3	2/17

Supplementary Table 2: 185 significantly altered genes from IDC versus NAT comparison with 99 validated genes from our second cohort, as well as the 95 validated genes from the TCGA cohort. The 66 genes that are significantly different in all three cohorts are shown in bold. 28 genes are highlighted with * are TNBC specific genes validated in our second cohort, 14 genes highlighted with ^ are TNBC specific genes validated in the TCGA cohort. NS = "not significant" in the validation/TCGA cohort; NOA = "Not on Array" that was used for validation/TCGA cohort.

		**Study cohort**		**Validation cohort**		**TCGA cohort**	
Gene type	Transcript ID	Fold-Change(IDC vs. NAT)	p-value(IDC vs. NAT)	Fold-Change (IDC vs. NAT)	p-value(IDC vs. NAT)	Fold-Change (IDC vs. NAT)	p-value(IDC vs. NAT)
**Protein coding transcripts**	AADACL2	-2.06	3.20E-06	-4.76	1.76E-10		NS
	**ABCA10**	-2.71	5.51E-10	-4.69	1.76E-09	-3.07	3.59E-21
	**ABCA6**	-3.83	4.95E-10	-5.01	6.37E-06	-8.96	5.42E-23
	**ABCA8**	-2.58	6.91E-10	-5.41	7.23E-05	-20.18	5.12E-29
	**ABCA9**	-2.54	4.50E-08	-4.55	2.43E-06	-15.16	2.85E-35
	**ABI3BP**	-4.32	9.39E-10		NS	-2.94	7.05E-14
	ADH1B *	-2.47	1.42E-06	-3.88	0.005398		NOA
	**AGR2 ***	-2.1	3.56E-06	-20.16	3.97E-05	-13.79	5.62E-49
	AGR3 *	-3.01	8.72E-12	-34.24	1.48E-15		NOA
	AMD1	2.32	3.92E-04		NS		NS
	**ANKRD30A *^**	-4.91	9.08E-12	-43.09	5.71E-12	-31.96	1.48E-25
	**ANKRD30B ***	-2.24	4.58E-04	-13.74	0.000141	-11.36	1.11E-17
	ANKRD36BP1 *	2.07	8.39E-04	3.44	0.000109		NOA
	**ANLN**	2.56	9.26E-06	15.88	2.62E-08	8.94	2.52E-56
	**ANP32E *^**	2.05	9.32E-04	2.58	2.37E-06	3.06	5.47E-40
	**APOD ***	-7.77	9.23E-09	-4.71	0.002126	-5.16	1.65E-16
	**AREG ***	-2.06	2.80E-06	-7.97	0.000308	-16.35	1.54E-12
	**ASPM**	2.97	4.08E-07	15.69	5.15E-08	19.80	2.17E-71
	**ATAD2**	2.63	2.13E-06	2.93	0.002838	3.95	3.05E-32
	**BGN**	2.48	1.21E-05	8.53	1.04E-05	3.38	1.20E-42
	C21orf91 ^	2.05	4.29E-05		NS	2.18	6.56E-37
	C3orf62	-2.02	4.69E-06		NS		NS
	**CALU**	2.19	3.88E-06	2.86	3.88E-08	3.26	1.72E-27
	CCDC90A	2.11	1.89E-04		NS		NOA
	CCL28	-2.14	9.17E-05	-7.42	0.003923		NS
	CCNA2	2.55	1.79E-06	5.72	3.44E-06		NOA
	**CCT3**	2.09	3.04E-06	2.39	1.16E-07	2.46	2.70E-42
	CD24 *	3.24	1.45E-05	1.94	0.003934		NS
	CD34	-2.07	1.14E-06		NS		NS
	CD36	-2.48	4.12E-07		NS	-12.09	4.36E-20
	**CENPF**	2.94	6.16E-08	14.06	1.36E-08	18.38	9.29E-69

	**CHML**	2.12	2.98E-05	3.72	1.11E-05	2.58	1.22E-19
	CHRDL1	-2.21	3.71E-07		NS	-3.14	1.96E-28
	**CKS2**	4.2	6.45E-08	4.93	2.83E-05	5.13	3.45E-56
	CLEC3B	-3.25	9.91E-11		NS	-7.34	1.12E-46
	CLEC7A	2.5	3.82E-06		NS	4.93	3.13E-17
	COL10A1	4.44	1.50E-07		NS	29.09	1.78E-109
	**COL14A1 ***	-3.44	5.00E-09	-3.01	0.000568	-5.57	4.10E-14
	CPE	-2.74	6.18E-09		NS	-7.20	7.49E-31
	**CXCL10**	4.48	1.19E-05	24.83	6.19E-06	3.09	8.20E-33
	**CXCL11**	2.03	6.14E-05	11.91	0.000101	9.68	9.14E-31
	CXCL12	-2.6	8.37E-09		NS	-2.69	6.40E-16
	**CXCL9**	5.14	1.96E-05	6.31	0.005079	3.20	6.32E-17
	DCLK1	-2.46	1.27E-08		NS		NOA
	DCN	-3.29	8.40E-10		NS	-5.58	1.40E-17
	DEK ^	2.01	2.76E-04		NS	1.82	8.99E-21
	DNAPTP3	6.06	2.17E-07	3.35	1.32E-05		NOA
	**DSC2 *^**	2.38	9.00E-04	1.89	0.006364	5.31	1.63E-29
	**ECT2**	2.52	4.48E-07	3.38	0.000117	4.47	2.82E-34
	EFEMP1	-2.13	2.58E-04		NS	-3.68	2.05E-17
	**EGR1**	-3.08	9.70E-07	-4.73	5.73E-05	-12.87	8.24E-38
	ESRP1	2.23	8.75E-06		NS		NOA
	FABP4	-3.61	8.74E-07		NS	-13.40	4.04E-15
	**FGF10 ***	-2.34	7.78E-11	-3.04	3.45E-05	-10.16	5.14E-16
	FGF7	-2.53	1.47E-04		NS	-3.67	4.15E-15
	**FN1**	3.94	1.26E-06	7.9	2.21E-12	4.73	3.88E-48
	**FREM1**	-2.18	9.51E-09	-2.37	7.86E-06	-13.48	2.05E-36
	GFPT1 ^	2.03	1.74E-04		NS	1.93	3.51E-11
	**GPI ^**	2.23	9.43E-06	3.66	0.000226	1.87	3.91E-09
	HBA1	-2.09	3.65E-05		NS	-3.92	1.33E-44
	HBA2	-2.37	4.60E-06		NOA	-5.71	1.45E-43
	HIST1H2AE	2.42	3.06E-05		NOA	2.43	4.63E-20
	HIST1H2AI *	2.24	7.53E-05	2	0.000112		NOA
	**HIST1H2BD**	2.47	5.20E-04	2.3	0.004617	2.60	1.71E-32
	HIST1H2BG	3.78	4.34E-05		NOA	2.71	1.67E-22
	HIST1H2BM	2.12	4.21E-04		NS	2.04	1.69E-22
	**HIST1H3F ***	4.64	8.42E-07	1.61	0.002149	2.65	1.06E-38
	HIST1H3I	3.15	2.38E-06		NS		NS
	HIST1H4L *	2.17	1.25E-03	1.75	0.006481		NS
	HIST2H2AB	3.13	2.90E-06		NS	3.11	2.89E-36
	HORMAD1 ^	3.28	4.46E-06		NS	20.62	3.09E-54
	HSPB6	-3.93	6.45E-12		NS	-2.07	2.13E-35

	**IGF1 ***	-2.96	1.21E-08	-3.65	0.000782	-5.83	1.42E-18
	IGHA2	-5.39	1.52E-07		NOA		NOA
	IGJ	-4.48	4.62E-06		NS		NS
	**IL6ST *^**	-2.36	7.53E-06	-3.39	8.60E-08	-3.20	1.92E-23
	**INHBA**	2.12	3.42E-06	7.48	1.49E-06	5.23	3.52E-60
	**JAM2**	-2.08	6.94E-08	-2.47	0.001	-4.20	2.88E-29
	**KIF11**	2.4	1.92E-06	8.69	1.39E-05	6.69	6.47E-60
	**KPNA2**	2.16	9.43E-05	5.28	4.32E-08	3.72	6.80E-37
	**LAMA2 ***	-2.02	7.43E-07	-2.51	0.001598	-4.28	4.96E-14
	LEP	-2.53	1.77E-06		NS		NS
	**LIFR**	-2.67	3.25E-07	-5.28	0.000688	-7.78	1.84E-43
	LRRC15	2.04	1.22E-04		NS	3.12	2.02E-33
	MAL2	2.19	1.31E-06		NS		NS
	**MELK**	2.13	2.71E-05	14.49	1.27E-07	14.34	1.72E-61
	**MKI67**	2.18	9.96E-06	10.94	5.24E-08	14.02	1.35E-51
	**MME**	-2.08	6.00E-05	-5.17	0.001052	-8.64	6.89E-40
	MUCL1	-3.17	1.34E-03	-16.47	0.000281		NOA
	**MYBL1 ***	2.24	7.28E-04	4.34	0.005912	5.34	8.72E-17
	MYH11	-2.59	1.38E-07	-4.97	9.52E-08		NS
	NDC80	2.42	8.30E-07		NOA		NOA
	**NUSAP1**	2.14	7.03E-07	8.42	1.55E-06	6.35	3.64E-46
	**OGN ***	-4.13	7.52E-11	-5.26	5.37E-05	-58.54	4.14E-32
	OR5P2	-2.41	4.42E-04	-6.33	1.40E-06		NS
	PCDH18	-2.29	6.78E-06		NS	-2.68	2.89E-14
	**PDK4**	-2.53	1.90E-03	-3.74	0.006427	-15.78	4.50E-36
	PI15	-2.07	1.23E-04	-17.34	0.000186		NS
	PIGR	-3.19	5.37E-07	-5.45	7.03E-05		NS
	PIK3R1	-2.03	7.37E-05	-2.41	0.000102		NS
	PIP *	-8.96	1.08E-06	-62.8	5.64E-07		NS
	**PRDX1**	2	2.67E-05	1.92	0.001102	1.91	8.91E-14
	**PRR11 ^**	2.3	1.58E-06	9.5	1.28E-08	2.52	2.88E-17
	**RAD51AP1**	2.2	7.80E-06	4.3	0.00132	4.44	1.47E-29
	RBM34	2.21	7.94E-07		NS		NS
	**RBMS3**	-2.17	3.32E-07	-2.82	0.00085	-5.25	2.31E-35
	RGS1	2.2	1.13E-04		NS		NS
	S100A9	2.76	9.01E-04		NS		NS
	SCGB1D2 ^	-4.04	1.19E-05		NOA	-7.04	4.25E-16
	SCGB2A2 *	-5.05	4.01E-05	-39.28	1.47E-06		NS
	SKA3	2	8.80E-06	4.47	0.000656		NOA
	SKIL	2.02	9.45E-05		NS	2.79	1.38E-24
	**SLC7A5 ***	2.09	2.09E-03	2.25	0.000817	3.96	2.13E-32

	**SMC4**	2.01	1.56E-05	2.7	7.86E-05	3.15	1.08E-37
	**SNRPD1 ^**	2.06	1.18E-04	2.39	0.000965	1.91	2.42E-26
	**SPARCL1 ***	-2.65	9.09E-06	-1.93	0.001856	-2.16	2.33E-13
	**SPP1**	3.4	2.30E-06	11.48	4.91E-06	6.68	2.97E-12
	SRP9	2.11	4.66E-04		NS		NS
	**STIL**	2.14	7.21E-06	4.23	9.29E-05	9.28	1.33E-38
	STK38L ^	2.25	3.86E-06		NS	1.89	1.87E-13
	**SULF1**	3.38	7.50E-08	5.76	2.21E-07	2.95	9.23E-24
	SYNGR2	2.02	7.77E-04		NS		NS
	**TAT**	-2.27	6.83E-06	-60.22	2.49E-09	-3.68	3.73E-15
	**TBC1D9 ***	-2.25	1.37E-07	-4.12	2.21E-07	-7.35	7.69E-50
	TFRC	2.06	2.14E-05		NS		NS
	TMEM144 *	-2.04	5.22E-07	-3.19	1.24E-06		NS
	TMEM65 ^	2.79	7.64E-05		NS	3.90	3.17E-30
	TMSB10	3.31	1.09E-08	2.54	0.000969		NS
	TMSB15A	2.48	8.05E-04		NS		NOA
	**TOP2A**	4.13	6.26E-08	15.44	4.91E-10	8.58	4.63E-44
	**TPX2**	3.57	6.46E-08	11.83	2.71E-09	11.10	2.56E-63
	**TSHZ2**	-2.28	2.10E-06	-4.92	3.56E-05	-3.71	3.89E-20
	**UBE2C**	2.22	1.53E-07	2.6	0.000417	19.93	8.26E-57
	**UBE2T**	2.18	2.82E-05	8.55	1.24E-07	6.22	5.05E-47
**Non-protein coding transcripts**	GNG5P2	2.52	2.18E-04		NOA		NOA
	KCNQ5-IT1	2.16	4.06E-04		NOA		NOA
	LINC00357	-2.18	1.52E-03		NOA		NOA
	LOC100132529	-3.49	4.19E-08		NOA		NOA
	LOC100287562	-2.19	1.39E-05		NOA		NOA
	LOC100506980	2.68	1.24E-04		NOA		NOA
	LOC100653008	-2.71	7.06E-05		NOA		NOA
	LOC100653151	2.93	1.19E-03		NOA		NOA
	LOC727896	2.03	6.69E-04		NOA		NOA
	**MEG3 ***	-2.53	6.64E-09	-1.66	0.003507	-3.39	3.52E-68
	MIR21	-2.07	6.26E-04	-6.45	9.40E-06		NOA
	MIR320D1	-2.78	9.67E-04		NOA		NOA
	MIR558	2.01	1.47E-03		NOA		NOA
	MIR99A	-2.22	1.41E-06	-1.62	6.37E-06		NOA
	MME-AS1	-2.67	1.09E-06		NOA		NOA
	RN5S303	-2.01	2.64E-04		NS		NOA
	RNU2-5P	-2.25	8.23E-05		NS		NOA
	RNU4-4P	2.35	1.54E-05		NS		NOA
	RNU5A-8P	3.39	1.29E-04		NS		NOA
	RNU5E-1	2.32	1.09E-03		NOA		NOA

	SCARNA9L	2.34	7.39E-04		NS		NOA
	SNORA11	-2.12	3.53E-04		NOA		NOA
	SNORA71D	2.36	1.31E-03		NS		NOA
	SNORD113-2	-4.2	1.48E-07		NOA		NOA
	SNORD113-3	-3.64	4.06E-08	-4.56	2.98E-05		NOA
	SNORD114-1	-5.22	3.99E-05		NOA		NOA
	SNORD114-11	-2.18	1.12E-04		NOA		NOA
	SNORD114-15	-2.14	9.23E-08		NOA		NOA
	SNORD114-19	-3.35	2.50E-06		NOA		NOA
	SNORD114-2	-5.2	2.54E-09	-5.93	6.86E-08		NOA
	SNORD114-21	-2.24	7.04E-06		NOA		NOA
	SNORD114-25	-2.64	4.34E-09		NOA		NOA
	SNORD114-28	-2.03	6.52E-06		NOA		NOA
	SNORD114-3	-3.85	5.71E-10	-1.86	0.000129		NOA
	SNORD114-7	-3.26	4.29E-07		NOA		NOA
	SNORD116-1	-3.13	5.43E-04	-2.92	9.75E-05		NOA
	SNORD116-15	-2.18	1.07E-03	-2.9	3.47E-09		NOA
	SNORD116-17	-2.83	6.91E-04		NOA		NOA
	SNORD116-2	-3.52	1.89E-04		NS		NOA
	SNORD116-20	-2.01	1.99E-03		NOA		NOA
	SNORD116-24	-4.11	4.93E-04	-2.69	1.05E-05		NOA
	SNORD116-3	-3.44	2.13E-04	-10.47	1.27E-06		NOA
	SNORD116-5	-3.05	1.07E-03	-8.73	4.85E-07		NOA
	SNORD116-6	-3.47	1.70E-04	-6.94	6.70E-06		NOA
	SNORD116-8	-3.52	1.42E-04	-11.37	1.43E-06		NOA
	SNORD3A	2.57	5.67E-06		NS		NOA
	SNORD3B-1	2.6	4.83E-06		NOA		NOA
	SNORD3C	2.22	1.41E-04		NOA		NOA
	SNORD41	2.3	1.56E-03		NS		NOA
	SNORD78	5.37	2.97E-04		NS		NOA
	TSIX	-2.38	2.09E-05		NS		NOA

Supplementary Table 3: Results from IPA Core Analysis of 185 significant genes (IDC vs NAT).

	Gene/disease/function	p-value	# Molecules	Consistency score
Upstream regulators	PTGER2	1.33E-16		
	ERBB2	6.21E-16		
	E2F4	3.58E-13		
	TGFB1	3.34E-12		
Diseases and disorders	Cancer	2.61E-14 - 6.83E-04	119	
	Organismal Injury and Abnormalities	2.69E-14 - 5.11E-04	88	
	Reproductive System Disease	2.69E-14 - 5.11E-04	82	
Molecular and Cellular Functions	Cell-to-Cell Signaling and Interaction	1.27E-12 - 5.69E-04	38	
	Cell Cycle	1.80E-11 - 6.83E-04	42	
	Cellular Movement	3.04E-11 - 6.83E-04	51	
	Cell Morphology	3.58E-09 - 4.56E-04	49	
	Cellular Development	7.68E-09 - 6.83E-04	60	
Physiological System Development and Function	Cell-mediated Immune Response	1.46E-08 - 4.44E-04	10	
	Immune Cell Trafficking	1.46E-08 - 5.69E-04	27	
	Tissue Morphology	4.43E-08 - 5.23E-04	41	
	Tissue Development	1.05E-07 - 5.00E-04	56	
Top Regulator Effect Networks	ADIPOQ, DOCK8, IL10, NR3C1, Tnf (family): cell death of liver cells (+4 more)			16.364
	ADIPOQ, HGF, NR3C1: development of epithelial tissue			2.846
	ADIPOQ: differentiation of cells			-5.307
Top Networks	Cancer, Gastrointestinal Disease, Hepatic System Disease			55
	Cell-To-Cell Signaling and Interaction, Cellular Movement, Hematological System Development and Function			26
	Cell Death and Survival, Hematological System Development and Function, Hematopoiesis			24
	Infectious Disease, Carbohydrate Metabolism, Molecular Transport			24
	Cellular Development, Cellular Growth and Proliferation, Connective Tissue Development and Function			23

# of molecules shows the number of molecules in the pathway that are from our list of 185 significant genes

A Consistency Score: higher scores mean networks are directionally consistent, meaning that most of the paths from regulator to target to disease/ function are consistent with the predicted state of the regulator, the observed direction of expression of the target in the dataset and the expected impact on the disease/function downstream, based on findings from the literature.

Supplementary Table 4: Clinical information of samples in the validation cohort

Sample identifier	Age at diagnosis	Grade	ER status	PR status	HER2 status	Tissue sample
1	42	3	Positive	Negative	Positive	IDC
2	31	3	Positive	Positive	Negative	IDC
3	61	3	Positive	Positive	Negative	IDC
4	83	2	Positive	Positive	Positive	IDC
5	41	3	Positive	Positive	Negative	IDC
6	46	1	Positive	Positive	Negative	IDC
7	51	3	Positive	Positive	Negative	IDC
8	45	3	Positive	Positive	Negative	IDC
9	46	3	Positive	Positive	Negative	IDC
		None	None	None	None	NAT
10	63	1	Positive	Positive	Negative	IDC
11	69	3	Positive	Positive	Negative	IDC
12	65	3	Negative	Negative	Negative	IDC
13	42	2	Positive	Positive	Negative	IDC
14	60	3	Negative	Negative	Positive	IDC
15	38	3	Positive	Positive	Negative	IDC
16	42	3	Positive	Positive	Negative	IDC
17	29	3	Positive	Positive	Positive	IDC
18	40	3	Positive	Positive	Negative	IDC
19	53	1	Positive	Positive	Negative	IDC
20	64	1	Positive	Positive	Negative	IDC
21	57	3	Positive	Negative	Positive	IDC
22	71	3	Negative	Negative	Negative	IDC
23	65	3	Negative	Negative	Positive	IDC
24	78	3	Negative	Negative	Negative	IDC
25	56	2	Positive	Positive	Negative	IDC
26	59	2	Positive	Positive	Negative	IDC
27	59	1	Positive	Positive	Negative	IDC
28	78	1	Positive	Positive	Negative	IDC
29	82	3	Negative	Negative	Negative	IDC
30	72	3	Positive	Positive	Negative	IDC
31	54	3	Negative	Negative	Positive	IDC
32	80	1	Positive	Positive	Negative	IDC
33	51	3	Negative	Negative	Negative	IDC
34	90	3	Positive	Positive	Negative	IDC
35	78	3	Negative	Negative	Positive	IDC
36	73	3	Positive	Negative	Positive	IDC
37	74	3	Negative	Negative	Negative	IDC
38	50	3	Negative	Negative	Negative	IDC
39	48	3	Negative	Negative	Negative	IDC

40	58	3	Negative	Negative	Negative	IDC
41	65	3	Positive	Positive	Negative	IDC
42	50	3	Negative	Negative	Negative	IDC
43	44	3	Positive	Negative	Negative	IDC
44	44	1	Positive	Positive	Negative	IDC
45	45	3	Negative	Negative	Negative	IDC
46	36	3	Negative	Negative	Positive	IDC
47	52	3	Positive	Positive	Negative	IDC
48	53	3	Negative	Negative	Positive	IDC
		None	None	None	None	NAT
49	78	2	Positive	Positive	Negative	IDC
50	51	3	Negative	Negative	Positive	IDC
51	44	1	Positive	Positive	Negative	IDC
		None	None	None	None	NAT
52	34	3	Negative	Negative	Negative	IDC
53	75	3	Negative	Negative	Negative	IDC
54	68	3	Negative	Negative	Positive	IDC
55	62	3	Negative	Negative	Negative	IDC
56	49	2	Positive	Positive	Negative	IDC
57	75	3	Negative	Negative	Positive	IDC
58	61	3	Negative	Negative	Positive	IDC
59	48	3	Negative	Negative	Negative	IDC
60	63	1	Positive	Positive	Negative	IDC
61	46	3	Negative	Negative	Negative	IDC
62	66	1	Positive	Positive	Negative	IDC
		None	None	None	None	NAT
63	52	3	Positive	Positive	Positive	IDC
64	41	3	Positive	Positive	Negative	IDC

Supplementary Table 5: Fold changes and p-values of 83 significantly altered genes in lymph node positive primary tumours and lymph node metastasis. Transcripts with grey background are non-protein coding transcripts all others are protein coding transcripts.

Transcript ID	Fold-Change(IDC+ vs. NAT+)	p-value(IDC+ vs. NAT+)	Fold-Change(LNmet vs. NAT+)	p-value(LNmet vs. NAT+)
**AADACL2**	-2.12	1.54E-05	-2.24	7.11E-06
**ABRACL**	2.38	1.23E-05	2.40	1.64E-05
**ACTR2**	1.94	1.54E-05	1.82	0.000112
**ADH1B**	-2.24	0.000331	-2.37	0.000193
**AGR3**	-3.31	1.92E-10	-2.87	1.44E-08
**AMIGO2**	-1.68	0.000167	-1.77	6.19E-05
**ANLN**	2.77	0.000103	2.77	0.000153
**APOD**	-6.62	3.03E-05	-9.46	2.33E-06
**ARL6IP1**	1.93	8.71E-05	1.87	0.000259
**BUB1**	1.90	0.000162	1.98	9.93E-05
**C5orf4**	-1.75	0.00013	-1.95	1.27E-05
**C7orf63**	-1.53	0.000255	-1.75	6.03E-06
**CCL14-CCL15**	-1.68	1.37E-05	-1.54	0.000265
**CCT3**	2.44	2.01E-05	2.13	0.000316
**CD34**	-1.94	0.000179	-2.20	2.10E-05
**CD36**	-2.67	7.37E-07	-2.57	2.77E-06
**CDCA2**	1.85	0.000332	1.90	0.000302
**CENPF**	2.87	1.26E-05	2.39	0.000315
**CENPI**	1.73	0.000356	1.86	9.66E-05
**CHML**	2.42	0.000165	2.38	0.000305
**CKS2**	4.85	1.09E-06	4.00	1.92E-05
**DESI2**	2.20	1.23E-05	1.97	0.000193
**DHX9**	1.82	4.12E-06	1.79	1.21E-05
**DNA2**	1.90	0.000323	2.42	3.41E-06
**EGR1**	-2.85	0.000158	-3.27	4.06E-05
**EMCN**	-1.56	0.000295	-1.88	1.90E-06
**FABP4**	-3.63	1.01E-05	-3.42	3.43E-05
**FAM96A**	1.78	7.16E-05	1.69	0.000349
**FHL1**	-1.65	7.65E-05	-1.59	0.000329
**GNG5P2**	3.30	5.21E-05	3.21	0.000108
**GPC3**	-1.64	0.000362	-1.73	0.000155
**GREB1**	-1.56	0.000116	-1.57	0.000129
**HIST1H2AE**	2.82	0.000157	3.06	7.70E-05
**HIST1H3F**	3.67	0.000791	4.54	0.000174
**HIST2H2AB**	3.42	3.06E-05	3.65	2.06E-05
**IGSF10**	-1.90	0.000717	-2.26	4.52E-05
**IL20RA**	-1.62	4.27E-05	-1.70	1.49E-05
**ITIH5**	-1.60	0.000356	-1.78	3.16E-05
**ITM2A**	-1.72	0.000428	-1.95	3.85E-05

**JAM2**	-2.00	3.76E-06	-2.22	3.96E-07
**KCNK1**	1.71	5.16E-05	1.65	0.000187
**KIF11**	2.32	0.000251	2.32	0.000341
**KLHDC1**	-1.88	5.82E-05	-1.84	0.000139
**KPNA2**	2.31	0.00068	2.53	0.000264
**LEP**	-3.34	2.14E-06	-2.95	2.38E-05
**LIFR**	-2.80	3.00E-06	-2.43	5.93E-05
**LOC201651**	-1.77	1.27E-05	-1.88	3.42E-06
**LOC284825**	-1.77	2.34E-05	-1.66	0.000184
**LRRC17**	-1.54	0.000405	-1.67	6.38E-05
**MAD2L1**	1.83	0.000124	1.90	8.02E-05
**MAZ**	1.52	1.89E-05	1.59	5.13E-06
**MEG3**	-2.65	3.68E-08	-2.80	1.79E-08
**MIR3689F**	-1.65	0.000533	-1.75	0.000178
**MIR3911**	-2.26	0.000878	-2.57	0.000199
**MIR497HG**	-1.80	6.41E-06	-1.68	6.55E-05
**MME**	-2.00	0.00057	-3.06	3.65E-07
**MME-AS1**	-2.82	3.98E-06	-3.24	5.81E-07
**MMP16**	-1.51	0.000204	-1.57	9.02E-05
**MRPL47**	2.02	4.12E-05	2.00	8.02E-05
**NDC80**	2.48	3.52E-05	2.20	0.000359
**NEK10**	-1.75	0.000144	-1.87	4.41E-05
**NOVA1**	-1.67	1.64E-05	-1.60	9.34E-05
**NUSAP1**	2.14	3.06E-05	2.11	6.03E-05
**PI15**	-2.07	0.000681	-2.40	8.56E-05
**PLSCR4**	-1.77	0.000447	-2.06	2.49E-05
**RBMS3**	-2.08	5.91E-05	-2.30	1.10E-05
**RELN**	-1.78	8.99E-05	-1.80	0.000112
**RMI2**	1.67	8.07E-05	1.63	0.000211
**RTN4RL1**	-1.50	0.000264	-1.57	8.47E-05
**SMS**	2.03	0.000107	2.00	0.000205
**SNORD114-1**	-5.90	0.000277	-6.99	0.000117
**SNORD114-28**	-2.21	1.90E-05	-2.04	0.000142
**SPRY2**	-1.58	0.000249	-1.60	0.000263
**STAT1**	2.12	3.67E-05	2.12	5.55E-05
**SVEP1**	-1.88	7.05E-06	-1.87	1.43E-05
**TMSB10**	4.48	1.82E-09	3.96	3.57E-08
**TNFSF13B**	1.84	3.73E-05	1.95	1.20E-05
**TOMM20**	2.03	5.65E-05	1.95	0.000174
**TOP2A**	5.08	5.08E-07	4.63	2.88E-06
**TPX2**	3.40	2.48E-05	3.17	9.17E-05
**TSHZ2**	-2.14	0.000131	-2.33	3.94E-05
**TTC6**	-1.95	2.13E-07	-1.72	2.17E-05
**UBE2C**	2.01	0.000275	2.08	0.000215

Supplementary Table 6: Results from IPA Core Analysis of 83 genes associated with lymph node metastases.

	Gene/disease/function	p-value	# Molecules	Consistency score
Upstream regulators	E2F4	3.58E-08		
	CDKN1A	3.72E-08		
	ERBB2	8.08E-08		
Diseases and disorders	Cancer	2.90E-09 - 1.08E-02	62	
	Organismal Injury and Abnormalities	2.90E-09 - 1.08E-02	41	
	Reproductive System Disease	2.90E-09 - 1.08E-02	40	
Molecular and Cellular Functions	Cell Cycle	6.00E-12 - 1.08E-02	22	
	Cellular Assembly and Organization	6.13E-08 - 1.08E-02	20	
	DNA Replication, Recombination, and Repair	6.13E-08 - 1.08E-02	16	
	Cell Death and Survival	1.12E-04 - 1.08E-02	26	
	Cellular Function and Maintenance	2.26E-04 - 1.08E-02	14	
Physiological System Development and Function	Reproductive System Development and Function	7.63E-05 - 7.19E-03	5	
	Embryonic Development	5.32E-04 - 1.08E-02	12	
	Tissue Development	3.72E-04 - 1.08E-02	16	
Top Networks	Cell Cycle, Cellular Assembly and Organization, DNA Replication, Recombination, and Repair			41
	Cell Cycle, Humoral Immune Response, Cellular Movement			39
	Developmental Disorder, Hereditary Disorder, Neurological Disease			36
	Cellular Development, Cellular Growth and Proliferation, Cancer			26
	Cell Cycle, Cardiovascular System Development and Function, Cellular Movement			4

# of molecules shows the number of molecules in the pathway that are from our list of 185 significant genes

A Consistency Score: higher scores mean networks are directionally consistent, meaning that most of the paths from regulator to target to disease/ function are consistent with the predicted state of the regulator, the observed direction of expression of the target in the dataset and the expected impact on the disease/function downstream, based on findings from the literature.

Supplementary Table 7: 22 microRNAs significantly altered in IDC+ and LNmets samples but not in IDC- samples.

Probeset ID	Fold-Change(IDC LN+ vs. NAT LN+)	p-value(IDC LN+ vs. NAT LN+)	Fold-Change(LN vs. NAT LN+)	p-value(LN vs. NAT LN+)
hsa-let-7a	-2.05	6.49E-07	-2.37	3.24E-08
hsa-miR-100	-2.67	9.94E-05	-2.11	4.05E-03
hsa-miR-101	-1.62	2.02E-03	-1.67	1.67E-03
hsa-miR-125b-2*	-1.61	1.60E-09	-1.74	7.54E-11
hsa-miR-1274a	1.99	4.93E-04	2.44	2.76E-05
hsa-miR-1274b	1.75	6.43E-03	2.48	5.00E-05
hsa-miR-1280	1.95	3.52E-05	2.02	3.45E-05
hsa-miR-1308	2.97	5.47E-06	2.88	2.24E-05
hsa-miR-130a	-2.30	7.94E-04	-2.50	4.77E-04
hsa-miR-132	-1.83	2.82E-07	-1.85	7.47E-07
hsa-miR-145*	-1.58	5.56E-06	-1.59	1.07E-05
hsa-miR-204	-1.81	1.38E-08	-1.76	1.83E-07
hsa-miR-205	-3.21	4.10E-03	-6.74	1.89E-05
hsa-miR-218	-1.63	7.80E-06	-1.60	3.82E-05
hsa-miR-26a	-1.91	2.89E-05	-2.12	5.18E-06
hsa-miR-26b	-1.67	2.48E-04	-1.64	7.09E-04
hsa-miR-299-5p	-1.82	1.33E-07	-1.82	5.02E-07
hsa-miR-337-5p	-1.73	5.90E-09	-1.87	4.78E-10
hsa-miR-34a	-1.64	4.12E-03	-1.97	2.42E-04
hsa-miR-379	-1.79	8.28E-11	-1.98	2.34E-12
hsa-miR-381	-1.51	1.52E-07	-1.64	5.41E-09
hsa-miR-663	1.80	8.16E-03	2.16	1.18E-03

Supplementary Table 8: Correlation of significant genes and microRNAs.

The expression values of significantly altered genes and microRNAs in lymph node positive tumours and lymph node metastases have been correlated to identify potential microRNA targets in the list of genes, using Partek Genomic Suite 6.6. The integration is based on two prediction software suites (TargetScan 6.2 and microcosm) as well as the expression values of the microRNAs and genes. Genes written in bold are predicted in both prediction software suites.

	Gene Symbol	MicroRNA Name	Pearson correlation coefficient	p-value(Pearson correlation)	Spearman's rank correlation coefficient	p-value(Spearman's rank correlation)
targetscan 6.2	ARL6IP1	hsa-miR-130a	-0.216	8.95E-02	-0.257	4.24E-02
	**CENPF**	hsa-miR-205	-0.383	1.97E-03	-0.464	1.28E-04
	**CKS2**	hsa-miR-26a	-0.536	5.99E-06	-0.582	5.66E-07
		hsa-miR-26b	-0.533	6.84E-06	-0.585	4.86E-07
	DNA2	hsa-miR-26b	-0.590	3.64E-07	-0.625	4.46E-08
		hsa-miR-26a	-0.550	3.00E-06	-0.587	4.30E-07
		hsa-let-7a	-0.513	1.73E-05	-0.471	9.60E-05
	EGR1	hsa-miR-132	0.580	6.21E-07	0.531	7.56E-06
	**GPC3**	hsa-miR-204	0.446	2.49E-04	0.382	2.03E-03
	GREB1	hsa-miR-26b	0.231	6.86E-02	0.262	3.82E-02
		hsa-miR-26a	0.318	1.11E-02	0.370	2.80E-03
		hsa-let-7a	0.351	4.85E-03	0.355	4.25E-03
		hsa-miR-381	0.537	5.61E-06	0.502	2.81E-05
	**ITIH5**	hsa-miR-299-5p	0.347	5.35E-03	0.292	2.02E-02
	**KCNK1**	hsa-miR-26a	-0.349	5.02E-03	-0.385	1.82E-03
		hsa-miR-26b	-0.214	9.29E-02	-0.309	1.37E-02
	**KIF11**	hsa-miR-101	-0.375	2.45E-03	-0.383	1.93E-03
		hsa-miR-381	-0.321	1.02E-02	-0.297	1.80E-02
	**KPNA2**	hsa-miR-26b	-0.553	2.55E-06	-0.614	8.74E-08
		hsa-miR-26a	-0.518	1.35E-05	-0.558	2.07E-06
	LIFR	hsa-miR-218	0.304	1.56E-02	0.342	6.05E-03
		hsa-miR-101	0.312	1.29E-02	0.314	1.21E-02
		hsa-miR-26a	0.368	3.02E-03	0.409	8.83E-04
		hsa-miR-26b	0.424	5.36E-04	0.399	1.20E-03
	MAZ	hsa-miR-34a	-0.434	3.76E-04	-0.442	2.85E-04
	**MME**	hsa-miR-218	0.182	1.55E-01	0.147	2.49E-01
		hsa-miR-204	0.457	1.64E-04	0.362	3.51E-03
	MMP16	hsa-miR-26b	0.324	9.49E-03	0.294	1.94E-02
		hsa-miR-26a	0.414	7.52E-04	0.394	1.38E-03
		hsa-miR-132	0.560	1.86E-06	0.379	2.16E-03
	NOVA1	hsa-miR-101	0.371	2.74E-03	0.296	1.84E-02

		hsa-miR-132	0.537	5.59E-06	0.395	1.37E-03
		hsa-miR-26a	0.542	4.49E-06	0.495	3.68E-05
		hsa-miR-26b	0.545	3.83E-06	0.542	4.55E-06
		hsa-let-7a	0.609	1.20E-07	0.466	1.19E-04
		hsa-miR-204	0.626	4.05E-08	0.562	1.63E-06
	PI15	hsa-miR-100	0.227	7.41E-02	0.176	1.67E-01
		hsa-miR-299-5p	0.331	8.01E-03	0.253	4.56E-02
		hsa-miR-381	0.369	2.93E-03	0.325	9.33E-03
	**RELN**	hsa-miR-218	0.179	1.60E-01	0.170	1.83E-01
		hsa-miR-34a	0.358	3.98E-03	0.387	1.74E-03
	RTN4RL1	hsa-miR-34a	0.235	6.34E-02	0.264	3.66E-02
	TOMM20	hsa-miR-379	-0.303	1.59E-02	-0.373	2.63E-03
	TSHZ2	hsa-miR-218	0.255	4.41E-02	0.262	3.81E-02
		hsa-miR-101	0.286	2.31E-02	0.264	3.69E-02
		hsa-miR-34a	0.438	3.25E-04	0.444	2.71E-04
		hsa-miR-381	0.589	3.88E-07	0.445	2.63E-04
		hsa-miR-132	0.619	6.42E-08	0.497	3.48E-05
microcosm	AADACL2	hsa-miR-125b-2*	0.600	2.01E-07	0.529	8.25E-06
	APOD	hsa-let-7a	0.493	4.11E-05	0.420	6.03E-04
	C5orf4	hsa-miR-101	0.268	3.35E-02	0.229	7.11E-02
	CCT3	hsa-miR-125b-2*	-0.435	3.73E-04	-0.422	5.70E-04
		hsa-let-7a	-0.409	8.69E-04	-0.384	1.91E-03
		hsa-miR-205	-0.251	4.75E-02	-0.293	1.99E-02
		hsa-miR-130a	-0.154	2.27E-01	-0.186	1.45E-01
	CD34	hsa-miR-218	0.403	1.06E-03	0.477	7.68E-05
		hsa-miR-337-5p	0.597	2.45E-07	0.534	6.71E-06
	CD36	hsa-miR-26a	0.408	9.05E-04	0.393	1.46E-03
		hsa-miR-125b-2*	0.605	1.49E-07	0.519	1.32E-05
	CDCA2	hsa-miR-204	-0.422	5.66E-04	-0.363	3.47E-03
	**CENPF**	hsa-miR-205	-0.383	1.97E-03	-0.464	1.28E-04
	CENPI	hsa-miR-101	-0.174	1.72E-01	-0.141	2.71E-01
	**CKS2**	hsa-miR-26a	-0.536	5.99E-06	-0.582	5.66E-07
		hsa-miR-26b	-0.533	6.84E-06	-0.585	4.86E-07
		hsa-miR-145*	-0.385	1.82E-03	-0.375	2.47E-03
		hsa-miR-130a	-0.302	1.63E-02	-0.345	5.63E-03
	EMCN	hsa-miR-379	0.507	2.22E-05	0.524	1.06E-05
		hsa-miR-125b-2*	0.583	5.22E-07	0.590	3.54E-07
	FAM96A	hsa-miR-26a	-0.451	2.11E-04	-0.469	1.07E-04
		hsa-miR-26b	-0.366	3.19E-03	-0.459	1.54E-04
		hsa-miR-381	-0.250	4.85E-02	-0.243	5.48E-02
	**GPC3**	hsa-miR-34a	0.283	2.48E-02	0.294	1.93E-02

		hsa-miR-204	0.446	2.49E-04	0.382	2.03E-03
	HIST1H3F	hsa-miR-337-5p	-0.515	1.60E-05	-0.438	3.29E-04
		hsa-miR-101	-0.454	1.85E-04	-0.412	7.95E-04
		hsa-miR-125b-2*	-0.393	1.44E-03	-0.399	1.18E-03
	IGSF10	hsa-miR-337-5p	0.604	1.62E-07	0.412	7.96E-04
	**ITIH5**	hsa-miR-299-5p	0.347	5.35E-03	0.292	2.02E-02
		hsa-miR-26a	0.417	6.67E-04	0.392	1.50E-03
		hsa-miR-26b	0.425	5.09E-04	0.416	7.07E-04
	**KCNK1**	hsa-miR-100	-0.237	6.11E-02	-0.268	3.37E-02
	**KIF11**	hsa-miR-101	-0.375	2.45E-03	-0.383	1.93E-03
		hsa-miR-381	-0.321	1.02E-02	-0.297	1.80E-02
	KLHDC1	hsa-miR-101	0.466	1.18E-04	0.431	4.19E-04
	**KPNA2**	hsa-miR-26b	-0.553	2.55E-06	-0.614	8.74E-08
		hsa-miR-26a	-0.518	1.35E-05	-0.558	2.07E-06
		hsa-let-7a	-0.443	2.76E-04	-0.414	7.45E-04
		hsa-miR-130a	-0.142	2.68E-01	-0.218	8.63E-02
	LRRC17	hsa-let-7a	0.415	7.17E-04	0.307	1.44E-02
		hsa-miR-125b-2*	0.474	8.72E-05	0.443	2.72E-04
	**MME**	hsa-miR-26b	0.311	1.33E-02	0.300	1.70E-02
		hsa-miR-26a	0.335	7.25E-03	0.373	2.60E-03
		hsa-miR-125b-2*	0.491	4.38E-05	0.436	3.56E-04
	MRPL47	hsa-let-7a	-0.376	2.38E-03	-0.315	1.18E-02
	NEK10	hsa-miR-299-5p	0.255	4.39E-02	0.152	2.33E-01
		hsa-miR-218	0.328	8.58E-03	0.247	5.06E-02
	**RELN**	hsa-miR-218	0.179	1.60E-01	0.170	1.83E-01
	SVEP1	hsa-miR-299-5p	0.418	6.60E-04	0.422	5.80E-04
		hsa-miR-26b	0.486	5.40E-05	0.530	7.96E-06
		hsa-miR-381	0.511	1.87E-05	0.455	1.79E-04
		hsa-miR-26a	0.536	6.02E-06	0.631	3.00E-08
		hsa-miR-145*	0.589	3.92E-07	0.566	1.32E-06
	TMSB10	hsa-miR-100	-0.393	1.45E-03	-0.455	1.76E-04
		hsa-miR-299-5p	-0.321	1.03E-02	-0.308	1.40E-02
	TTC6	hsa-miR-130a	0.353	4.53E-03	0.371	2.77E-03
		hsa-miR-337-5p	0.520	1.28E-05	0.391	1.53E-03
		hsa-miR-145*	0.556	2.24E-06	0.403	1.05E-03
	UBE2C	hsa-miR-337-5p	-0.477	7.72E-05	-0.406	9.58E-04
		hsa-miR-379	-0.475	8.29E-05	-0.417	6.68E-04
		hsa-miR-381	-0.411	8.09E-04	-0.333	7.58E-03

Table 9: List of our miRNAs of interest that have been associated with TNBC or tumour progression previously

microRNA	What is known about this microRNA in TNBC	Reference
miR-101	Activates Akt and estrogen-independent growth	[1]
miR-145	Is down-regulated in TNBC<br>Is an onco-miR, targets BRCA1; effects BRCA1-mediated proliferation and homologous recombination	[2]<br>[3]
miR-125	Is down-regulated in TNBC and associated with chemo-resistance<br>Targets HER2	[4]<br>[5]
miR-130a	Is down-regulated in TNBC and associated with chemoresitance<br>Is a marker for HER2	[4]<br>[6]
miR-205	Is down-regulated in TNBC<br>Is a tumour-suppressor-miR; targets E2F1, LAMC1; suppresses proliferation, cell cycle and tumour growth<br>Lower levels are associated with lymph node metastasis	[2]<br>[7]<br>[8]
miR-34a	Is a tumour-suppressor-miR; targets AXL; inhibits migration	[9]
miR-381	Is associated with distant free survival and prognosis	[10]
let-7a	Is down-regulated in TNBC; tumour-suppressor-miR; targtes oncogenes including RAS, MYC, HMGA2	[11,12]

Supplementary Fig. 1: Unsupervised hierarchical clustering of 66 validated genes (common to study, second and TCGA cohort) significantly altered genes comparing IDC vs NAT samples.

Tumour vs normal comparison in our study cohort (33 IDC, 15 NAT), our second cohort (16 IDC, 4 NAT) and the TCGA TNBC cohort (55 IDC, 5 NAT) revealed 66 genes commonly differentially expressed between tumour and normal samples. A) Hierarchical clustering of 185 significantly altered genes in the TNBC study cohort. B) Hierarchical clustering of 66 significantly altered and validated genes in the TNBC study cohort. C) Hierarchical clustering of 66 significantly altered genes in our second cohort. D) Hierarchical clustering of 66 significantly altered genes in the TCGA TNBC cohort. TvN stands for the comparison of tumour vs normal samples. All normal adjacent samples (NAT) are shown in green and all primary tumour samples are shown in blue in the sample trees on the left (y-axis). Branches containing normal samples are shown in green, tumour samples in black. Genes are clustered along the x-axis. Low gene expression is shown in blue, high expression is shown in red and equivocal expression is shown in grey.

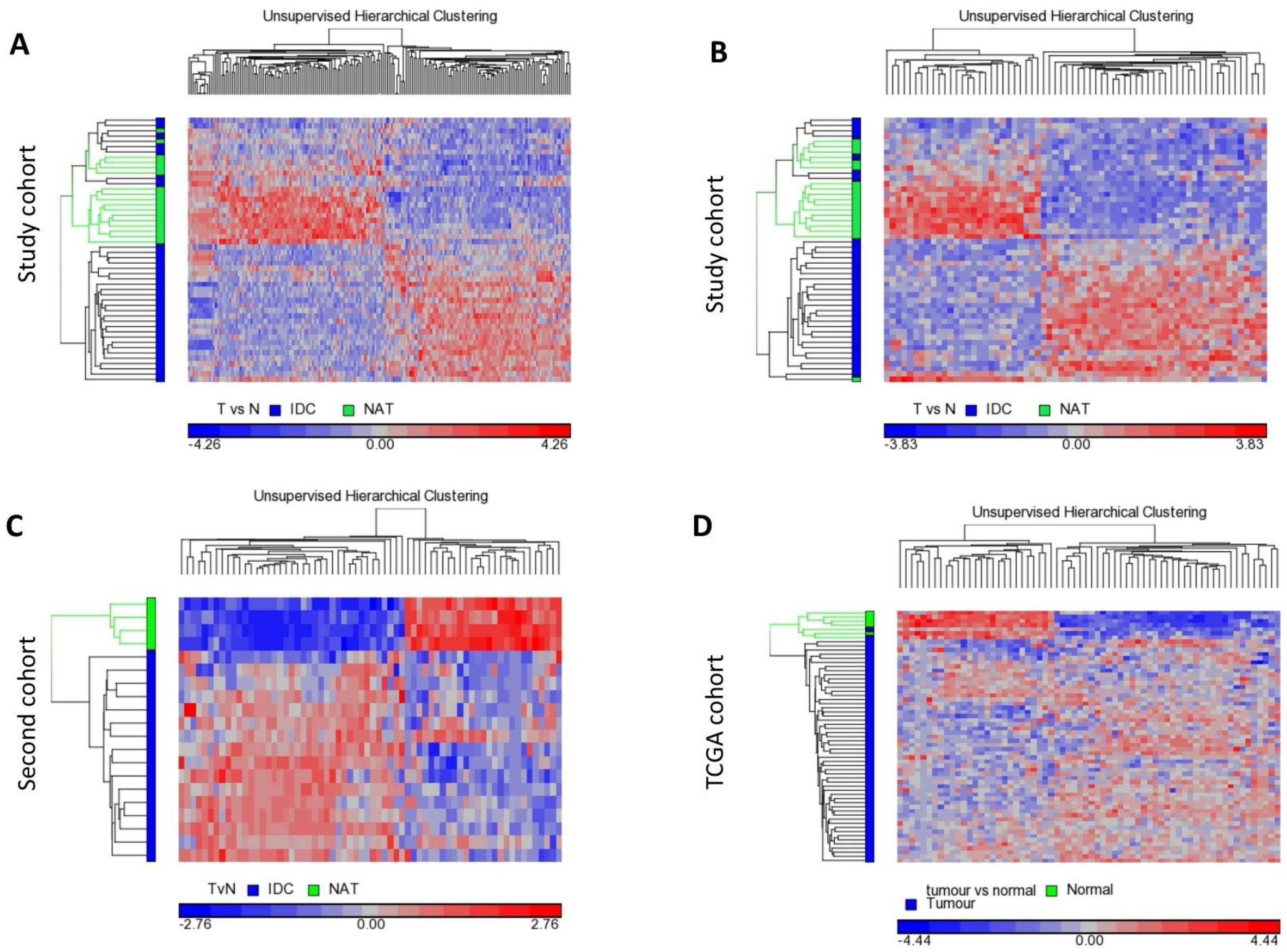
A
Unsupervised Hierarchical Clustering
Study cohort
T vs N
IDC
NAT
-4.26
0.00
4.26
B
Unsupervised Hierarchical Clustering
Study cohort
T vs N
IDC
NAT
-3.83
0.00
3.83
C
Unsupervised Hierarchical Clustering
Second cohort
TvN
IDC
NAT
-2.76
0.00
2.76
D
Unsupervised Hierarchical Clustering
TCGA cohort
tumour vs normal
Normal
Tumour
-4.44
0.00
4.44

Supplementary Figure 2: Expression levels in TNBC samples for estrogen receptor (ER), progesterone receptor (PR), and human epidermal growth factor receptor 2 (HER2) compared to receptor positive patients. The relative gene expression of all three receptors from TNBC samples has been compared to the expression of receptor positive patients from our second cohort. All genes were normalised to beta-2-microglobulin (B2M) as a housekeeping gene. Boxes represent the median ± interquartile range. **** p<0.0001; ** p= 0.001 to 0.01. Numbers in brackets represent the number of patients within the subgroups.

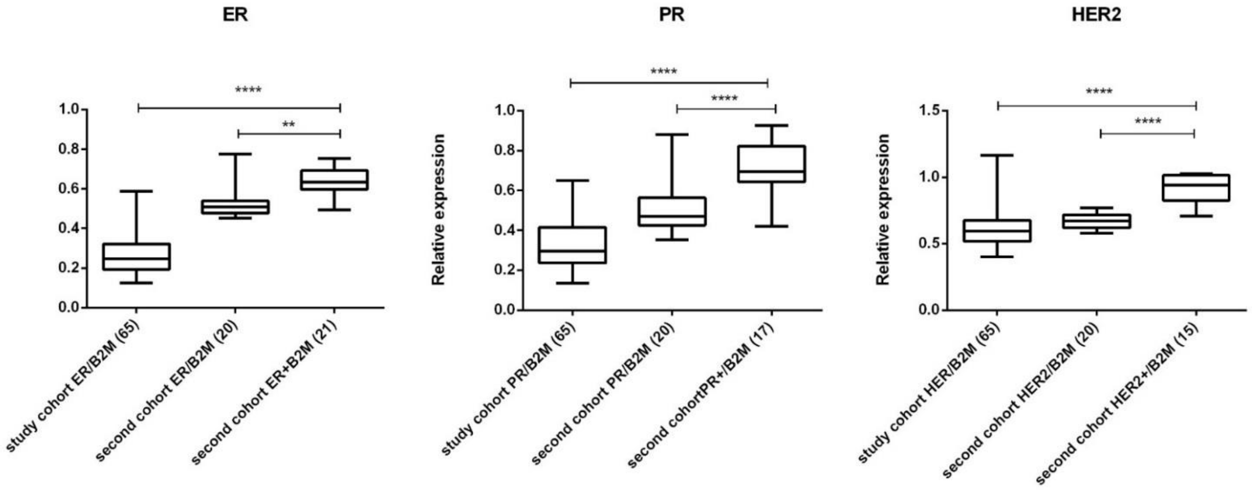

References

1. Sachdeva, M. *et al.* MicroRNA-101-mediated Akt activation and estrogen-independent growth. *Oncogene* **30**, 822-31 (2011).
2. Sempere, L.F. *et al.* Altered MicroRNA expression confined to specific epithelial cell subpopulations in breast cancer. *Cancer Res* **67**, 11612-20 (2007).
3. Garcia, A.I. *et al.* Down-regulation of BRCA1 expression by miR-146a and miR-146b-5p in triple negative sporadic breast cancers. *EMBO Mol Med* **3**, 279-90 (2011).
4. Ouyang, M. *et al.* MicroRNA profiling implies new markers of chemoresistance of triple-negative breast cancer. *PLoS One* **9**, e96228 (2014).
5. Scott, G.K. *et al.* Coordinate suppression of ERBB2 and ERBB3 by enforced expression of micro-RNA miR-125a or miR-125b. *J Biol Chem* **282**, 1479-86 (2007).
6. Lowery, A.J. *et al.* MicroRNA signatures predict oestrogen receptor, progesterone receptor and HER2/neu receptor status in breast cancer. *Breast Cancer Res* **11**, R27 (2009).
7. Piovan, C. *et al.* Oncosuppressive role of p53-induced miR-205 in triple negative breast cancer. *Mol Oncol* **6**, 458-72 (2012).
8. Berber, U. *et al.* miR-205 and miR-200c: Predictive Micro RNAs for Lymph Node Metastasis in Triple Negative Breast Cancer. *J Breast Cancer* **17**, 143-8 (2014).
9. Mackiewicz, M. *et al.* Identification of the receptor tyrosine kinase AXL in breast cancer as a target for the human miR-34a microRNA. *Breast Cancer Res Treat* **130**, 663-79 (2011).
10. de Rinaldis, E. *et al.* Integrated genomic analysis of triple-negative breast cancers reveals novel microRNAs associated with clinical and molecular phenotypes and sheds light on the pathways they control. *BMC Genomics* **14**, 643 (2013).
11. Bussing, I., Slack, F.J. & Grosshans, H. let-7 microRNAs in development, stem cells and cancer. *Trends Mol Med* **14**, 400-9 (2008).
12. Boyerinas, B., Park, S.M., Hau, A., Murmann, A.E. & Peter, M.E. The role of let-7 in cell differentiation and cancer. *Endocr Relat Cancer* **17**, F19-36 (2010).
